# Homogenized multiscale modelling of an electrically active double poroelastic material representing the myocardium

**DOI:** 10.1007/s10237-025-01931-0

**Published:** 2025-02-26

**Authors:** Laura Miller, Raimondo Penta

**Affiliations:** 1 School of Mathematics and Statistics, University of Glasgow, University Place, Glasgow, G12 8QQ UK; 2 Department of Mathematics and Statistics, University of Strathclyde, Richmond Street, Glasgow, G1 1XH UK

**Keywords:** Poroelasticity, Asymptotic homogenization, Myocardial modelling, Composite materials

## Abstract

In this work, we present the derivation of a novel model for the myocardium that incorporates the underlying poroelastic nature of the material constituents as well as the electrical conductivity. The myocardium has a microstructure consisting of a poroelastic extracellular matrix with embedded poroelastic myocytes, i.e. a double poroelastic material. Due to the sharp length scale separation that exists between the microscale, where the individual myocytes are clearly resolved from the surrounding matrix, and the length of the entire heart muscle, we can apply the asymptotic homogenization technique. The novel PDE model accounts for the difference in the electric potentials, elastic properties as well as the differences in the hydraulic conductivities at different points in the microstructure. The differences in these properties are encoded in the coefficients and are to be computed by solving differential cell problems arising when applying the asymptotic homogenization technique. We present a numerical analysis of the obtained Biot’s modulus, Young’s moduli as well as shears and the effective electrical activity. By investigating the poroelastic and electrical nature of the myocardium in one model, we can understand how the differences in elastic displacements between the extracellular matrix and the myocytes affect mechanotransduction and the influence of disease.

## Introduction

The human heart is a muscular organ comprising four chambers: two atria and two ventricles, each of which have a muscular wall with three distinct layers, the endocardium, the myocardium and the epicardium. The thin inner and outer layers are known as the endocardium and epicardium, whereas the myocardium is the middle, thickest and dominant layer. The myocardium has its own blood supply via the coronary arteries. The myocardium can be affected by a variety of diseases that influence its contractility and in turn its efficiency at pumping blood around the entire body, e.g. myocardial infarction, angina and the effects of ageing (Whitaker 2014; Weinhaus and Roberts 2005).

The myocardium comprises individual cardiac muscle cells that are connected together creating a functional electrical syncytium. The syncytium allows for the rapid yet coordinated contraction of the muscles cells along the entire length. Therefore, there exists strong electrical and mechanical interactions taking place in every direction between each of the adjacent cardiac muscle cells. These connections allow the myocardium to behave as a single contractile unit (Bader et al. 2021). These cardiac muscle cells are called myocytes, and the ends are connected by the gap junctions at the intercalated discs. Blood flow around the body is propelled by contraction of the heart muscle due to the electrical activation of the myocytes. The heart has a vast and complicated physiology and electrical conductivity, and we encourage the reader to consider (Katz 2010; Opie 2004; Weidmann 1974) to gain a more thorough understanding.

The electrophysiology and mechanical behaviour of the heart have been the subject of much investigation (Peirlinck et al. 2021; Owen et al. 2018; Smith et al. 2004). Key approaches in the literature surround the use of constitutive nonlinear elastic modelling via both the Holzapfel–Ogden law (Holzapfel and Ogden 2009) and Fung (2013). The Holzapfel–Ogden law takes into consideration the fibre orientations when modelling the layers of the myocardium. Other works focusing on the influence of microstructural fibre arrangements in both soft tissues and porous media are Federico and Herzog (2008); Hashlamoun et al. (2016). The Holzapfel–Ogden law has been used for a variety of studies such as the inclusion of residual stress (Wang et al. 2014) and parameter inference (Gao et al. 2015). Other important heart modelling approaches include the models of active contraction by Guccione et al. (1993); Guccione and McCulloch (1993) and the finite element models of ventricular mechanics and large-scale beating heart in Guccione and McCulloch (1991); Guccione et al. (2020). The theories of Fung have been applied to heart modelling in Fung (1970), and in Hsu et al. (2015), the authors use fluid–structure interaction simulations to understand bioprosthetic heart valves. A viscoelastic approach to modelling the myocardium has also been investigated by Gültekin et al. (2016) and Nordsletten et al. (2021). In Zingaro et al. (2023), the authors create a first of its kind computational model for myocardial blood perfusion that accounts for multiscale and multiphysics features. There have been many prominent works modelling the perfusion of the heart from a computational viewpoint. These include computational methods to obtain myocardial coronary permeabilities using the underlying anatomical vascular network, developed by Hyde et al. (2014). In Di Gregorio et al. (2021), the authors develop a mathematical and numerical model of cardiac perfusion accounting for the different length scales of the vessels in the coronary tree. Finally in Montino Pelagi et al. (2024), simulation of coronary flow and 3D myocardial perfusion is investigated.

The heart is highly multiscale in nature with various structural components only visible at various microstructural levels or scales. We are studying the myocardium where we wish to consider the interactions between the myocytes and the extracellular matrix. We therefore consider a length scale of resolution of the microstructure where we can visibly see the myocytes and matrix distinctly resolved from each other. This scale will be our microscale. This is a fine microstructural level of the myocardium and therefore can be characterized by a length which is much smaller than the one of the whole heart muscle. The complete heart muscle has a scale which we call the macroscale. We should note that while we will not resolve the interactions here in this work, if we zoom in further on each of the microscale components we find that each is a porous matrix with fluid flow in the pores, and this scale is known as the porescale. A porous media approach to modelling perfusion in soft tissues is taken by Huyghe and Van Campen (1995a, b), and in particular, modelling the myocardium and complex fluid flows has been considered in Cookson et al. (2012); Ng et al. (2005); Pesavento et al. (2017); Miller and Penta (2024). We note that although regularly not considered, it is a valid modelling assumption to consider the cardiac myocytes as a poroelastic material for two specific reasons. Firstly, all biological cells can be considered poroelastic due to the microscale existence of cytoplasm and various organelles (Moeendarbary et al. 2013), and secondly, the homeostasis of myocardial fluid content is controlled via the interplay between cardiac myocyte water uptake, microvascular filtration, interstitial hydration and lymphatic removal (Vasques-Nóvoa et al. 2022).

Since the heart comprises multiple scales, it is necessary to create computationally feasible models that can effectively characterize the effective behaviour of the organ. Such a model requires that the macroscale governing equations have the properties and interactions of the microscale encoded. To address this, a problem is setup that possesses governing equations for each phase in the microstructure and the interactions that occur between them. This type of problem can then undergo an upscaling process which will lead to a macroscale system of governing equations. This upscaling process can be carried out by a variety of methods each of which has been discussed in the literature and is collectively known as homogenization techniques. These techniques include volume averaging, mixture theory, effective medium theory and asymptotic homogenization. Homogenization techniques such as those mentioned above have been reviewed and discussed in Hori and Nemat-Nasser (1999); Davit et al. (2013).

We will utilize the two-scale asymptotic homogenization technique. This technique has gained popularity in the modelling of poroelasticity such as in Burridge and Keller (1981); Penta et al. (2020), in the modelling of elastic composites (Penta and Gerisch 2015; Penta and Gerisch 2017; Ramírez-Torres et al. 2018) and also in electroactive materials (Di Stefano et al. 2020; Penta et al. 2018; Penta et al. 2021). The theory of poroelasticity has been extended via the asymptotic homogenization so as to include important biological features such as growth and remodelling and vascularization of poroelastic materials (Penta et al. 2014; Penta and Merodio 2017). More recently, the technique was used to investigate poroelastic materials with more complicated microstructures such as poroelastic composites and double poroelastic materials (Miller and Penta 2020; Miller and Penta 2021a). A key feature of the asymptotic homogenization technique is that it produces computationally feasible models. Due to this, a variety of analyses have been carried out including a micromechanical analysis of the effective stiffness of poroelastic composites in Miller and Penta (2023) and the role of porosity and solid matrix compressibility on the mechanical behaviour of poroelastic tissues has been investigated in Dehghani et al. (2018). Asymptotic homogenization has been previously applied to solve problems in heart modelling. In Miller and Penta (2022), the structural changes involved in myocardial infarction have been investigated numerically. The technique has also been used in the context of the electrical bidomain model (see Bader et al. 2021; Richardson and Chapman 2011) and both the electrical and mechanical bidomain model of the heart (Miller and Penta 2023).

We apply the two-scale asymptotic homogenization technique to the equations we have chosen to govern the electrostatic and poroelastic interactions of the components of the myocardium. We are investigating the myocardium at a scale of resolution where the myocytes are distinctly visible from the matrix. We call this scale the microscale. We associate a length with the microscale that is much smaller than the length of the entire heart muscle. When looking at the entire heart muscle, we no longer see the myocytes as the variations are smoothed out, and so, we denote the scale of the heart as the macroscale. At a finer scale of resolution on both the myocytes and the extracellular matrix, we find that each domain comprises a porous matrix with fluid flowing in the pores. This porescale microstructure is captured via the use of the governing equations of Biot’s poroelasticity in each domain of the microscale. We are then able to apply the asymptotic homogenization technique to upscale the microscale problem, by accounting for the continuity of current densities, stresses, elastic displacements, fluxes, pressures and then also the difference in the electric potentials across the interface between the myocyte and the matrix. We note that an important feature of the asymptotic homogenization technique is that the scales and features fully decouple (see Penta and Gerisch 2017; Auriault et al. 2010; Mei and Vernescu 2010). The obtained novel system of macroscale PDEs contains balance equations for the current densities and the stresses, a conservation of mass equation and a modified Darcy’s law. The macroscale equations have coefficients which encode properties of the material microstructure, and these are calculated by solving the microscale cell problems (electric, poroelastic and Darcy flow) that arise from our upscaling. A key feature of the asymptotic homogenization is the decoupling of the scales which allows for straightforward solution of the cell problems, and we note that the physical features fully decouple. That is, we have cell problems for the electric features that are fully independent from the poroelastic problems, which are also fully independent from the Darcy flow problems.

The current work extends upon exciting modelling developments in the literature such as the electrical and mechanical bidomain model of Miller and Penta (2023), the models of a poroelastic matrix with elastic inclusion by Royer et al. (2019) and Chen et al. (2020), and the model of double poroelastic materials (Miller and Penta 2021a). Here we will combine key features from Miller and Penta (2023) and Miller and Penta (2021a) to create an electrical and mechanical myocardium model where there is an extra level of microstructural complexity encoded by accounting for the fact that the myocytes and matrix have an underlying poroelastic nature. This means we have the behaviour of two finer scales encoded in the macroscale model, and additionally, this allows for a greater understanding of the myocardial behaviour as we are using a more realistic microstructure. The key novelties of the model are that (1) the macroscale coefficients encode the differences in microstructure over two finer scales of resolution and (2) it encodes the difference in poroelastic and electrical properties/moduli at different points in the microstructure. Our macroscale stress balance equation captures how the elastic displacement of the myocyte and extracellular matrix are driven by the applied magnetic fields. Since our model captures both elastic and electrostatic activity, it paves the way towards understanding whether the differences in myocyte and extracellular matrix displacements affect the mechanotransduction of the overall heart and hence greater understanding of the influence of disease.

The paper is organized as follows. Section 2 introduces the mechanical and electrostatic equations that govern the interactions between the poroelastic myocyte and the poroelastic extracellular matrix. In Sect. 3, we apply a multiscale analysis to the problem to allow us to derive the new macroscale myocardial model encoding both the electrostatic and poroelastic properties of the myocardium. In Sect. 4, we present the new macroscale model and discuss the novel terms that arise. In Sect. 5, we solve the novel cell problems and present an analysis of both the effective electrical conductivity tensor and poroelastic properties such as elastic shears and Young’s moduli, as well as investigating the Biot’s modulus. Finally in Sect. 6, we summarize our work discuss limitations and provide further perspectives.

**Fig. 1:**
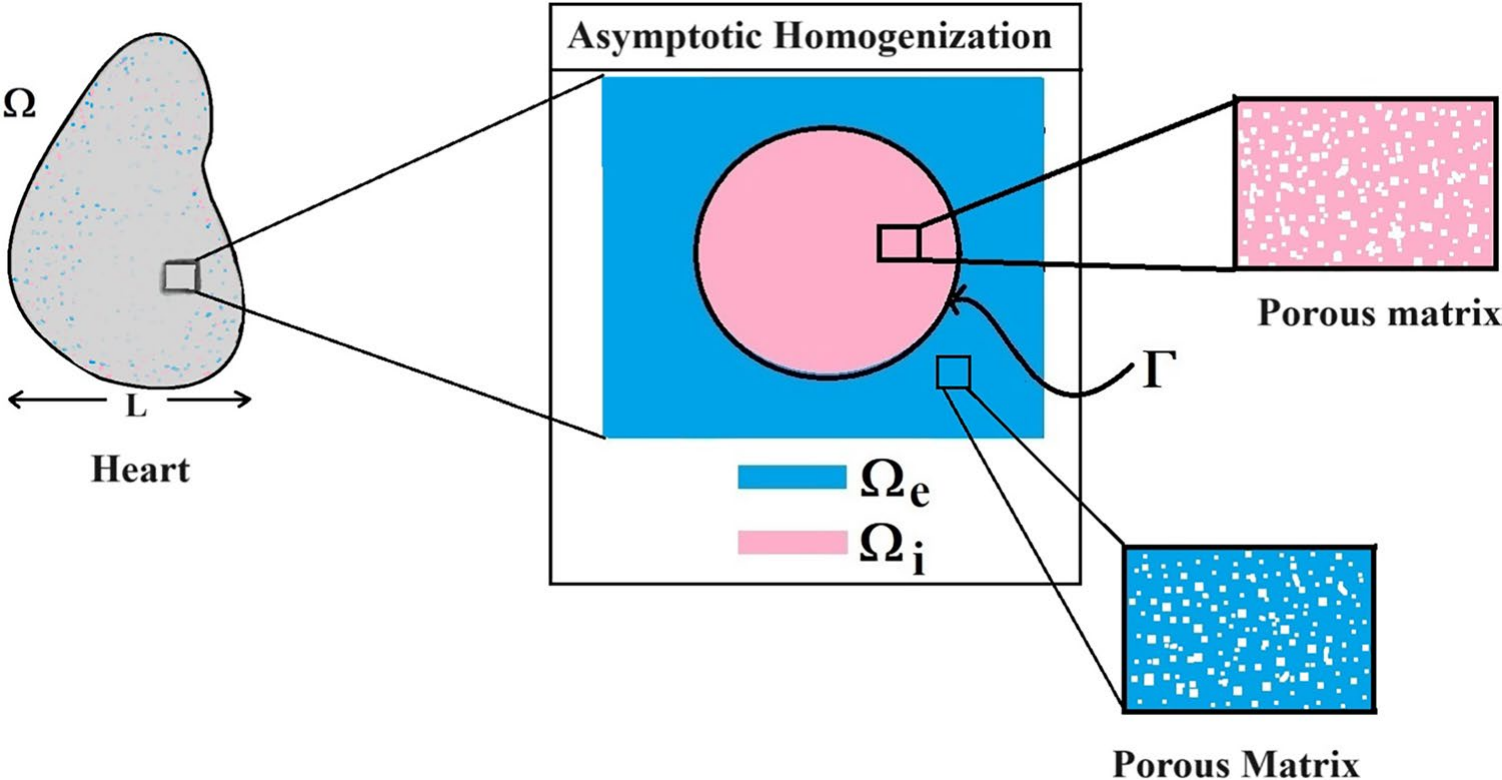
We have a 2D sketch representing a cross section of the 3D domain $\Omega$. The myocyte $\Omega _i$ is shown in pink and the extracellular domain $\Omega _e$ is in blue. There is an interface $\Gamma$ (the cell membrane) between the two domains. We also can see that when zooming in on each of the two domains we have two different porous media with a fluid flowing in the pores

## Problem

We consider the myocardium to be a set $\Omega \in {\mathbb {R}}^3$ where $\Omega$ is the union of the poroelastic extracellular matrix $\Omega _e$ and the poroelastic myocyte $\Omega _i$, where we can write ${\bar{\Omega }}={\bar{\Omega }}_i\cup {\bar{\Omega }}_e$. We provide a sketch of a cross section of the domain $\Omega$ in Fig. 1.

To create our model, we begin by writing the equations that govern each domain as well as the appropriate interface conditions that will close the problem, which we note will be in a quasi-static and linearized regime. We first consider the electrical component of our model and use the steady-state electrical bidomain equation, proposed by Puwal and Roth (2010); Roth (1992, 2016), in each subphase

1a $$\begin{aligned}&\nabla \cdot ({\textsf{G}}_{i}\nabla \phi _i)=\beta G(\phi _i-\phi _e) \qquad \textrm{in}\quad \Omega _i, \end{aligned}$$

1b $$\begin{aligned}&\nabla \cdot ({\textsf{G}}_{e}\nabla \phi _e)=-\beta G(\phi _i-\phi _e) \qquad \textrm{in}\quad \Omega _e, \end{aligned}$$

where we have the second rank conductivity tensors ${\textsf{G}}_{i}$ and ${\textsf{G}}_{e}$ in the myocyte and extracellular domain, respectively, and the scalar electric potentials of each phase are $\phi _i$ and $\phi _e$. We also have the membrane parameters: $\beta$ is the ratio of membrane area to tissue volume and *G* is the membrane conductance. Equations (1a) and (1b) are the balance equations for the electric current densities for each phase. We note that it would also be possible to assume that we do not have a passive membrane current and modify Eqs. (1a) and (1b) to include an active membrane current such as the model presented by Spach (1983). We further remark that we could include the addition of active stresses and active strain such as in Pezzuto and Ambrosi (2014); Pezzuto et al. (2014) to incorporate the heart actively beating and undergoing deformation. The current densities are given by Ohm’s law

2a $$\begin{aligned}&\textbf{j}_i=-{\textsf{G}}_{i}\nabla \phi _i \qquad \textrm{in}\quad \Omega _i, \end{aligned}$$

2b $$\begin{aligned}&\textbf{j}_e=-{\textsf{G}}_{e}\nabla \phi _e \qquad \textrm{in}\quad \Omega _e, \end{aligned}$$

where again the conductivity tensors are ${\textsf{G}}_{i}$ and ${\textsf{G}}_{e}$ and the applied electric fields are $\nabla \phi _i$ and $\nabla \phi _e$.

We also need to consider the poroelastic component of our model. The two phases need a balance equation that will govern their mechanical behaviour. These are given by

3a $$\begin{aligned}&\nabla \cdot {\textsf{T}}_i= \textbf{b}_i \qquad \textrm{in}\quad \Omega _i, \end{aligned}$$

3b $$\begin{aligned}&\nabla \cdot {\textsf{T}}_e= \textbf{b}_e \qquad \textrm{in}\quad \Omega _e. \end{aligned}$$

The stress tensors ${\textsf{T}}_i$ and ${\textsf{T}}_e$ are the stress tensors in each compartment and are those of linear poroelastic materials (Burridge and Keller 1981; Penta et al. 2020). We assume that each of the domains is subject to a body force $\textbf{b}_i$ and $\textbf{b}_e$. The body forces are the magnetic Lorentz force and are given by the action potential currents, $\textbf{j}_i$ and $\textbf{j}_e$, in a magnetic field $\textbf{B}$, as in Puwal and Roth (2010). The body forces $\textbf{b}_i$ and $\textbf{b}_e$ are therefore

4 $$\begin{aligned} \textbf{b}_i=\textbf{j}_i \times \textbf{B} &=   -{\textsf{G}}_i\nabla \phi _i\times \textbf{B} \, \text{ and }\, \textbf{b}_e=\textbf{j}_e \times \textbf{B}\nonumber \\&=  -{\textsf{G}}_e\nabla \phi _e\times \textbf{B}. \end{aligned}$$

The body forces chosen here are commonly selected in the literature (see Puwal and Roth 2010) when investigating the cardiac action potentials. We also apply this body force because there are important applications where the magnetic Lorentz force has been used in medical imaging, e.g. elastic displacement due to Lorentz force has been recently proposed as a potential use of MRI (Roth et al. 2014). For readers interested in electroelastic or magneto-elastic materials and applied electric body forces for a variety of modelling applications, see, for example, Dorfmann and Ogden 2006; Maugin 2013; Dorfmann and Ogden 2014; Fu 2024; Liguori and Gei 2023; Bustamante et al. 2009; Bustamante 2010.

We assume that the two solid phases can be described using Biot’s anisotropic, heterogeneous, compressible poroelasticity (Biot 1955; Biot 1956a, b; Biot 1962). This can be derived via application of the asymptotic homogenization technique to a finer scale problem where the solid phases are described using Cauchy stress tensor (see Burridge and Keller 1981; Penta et al. 2020). Therefore, we have the stresses ${{\textsf{T}}}_{i}$ and ${{\textsf{T}}}_{e}$ appearing in (3a) and (3b) which are given by

5a $$\begin{aligned}&{{\textsf{T}}}_{i}={\mathbb {C}}_{i}:\zeta (\textbf{u}_{i})-{\varvec{\alpha }}_{i}p_{i} \qquad \text{ in } \quad \Omega _{i}, \end{aligned}$$

5b $$\begin{aligned}&{{\textsf{T}}}_{e}={\mathbb {C}}_{e}:\zeta ( \textbf{u}_{e})-{\varvec{\alpha }}_{e}p_{e} \qquad \text{ in } \quad \Omega _{e}, \end{aligned}$$

where

6 $$\begin{aligned} \zeta (\cdot)=\frac{\nabla (\cdot)+(\nabla (\cdot))^{\textrm{T}}}{2}, \end{aligned}$$

is the symmetric part of the gradient operator. We have that $\textbf{u}_{i}$ and $\textbf{u}_{e}$ are the elastic displacements in the myocyte and the extracellular matrix, respectively, and $p_{i}$ and $p_{e}$ are the pressures in the myocyte and extracellular matrix, respectively. The ${\mathbb {C}}_{i}$ and ${\mathbb {C}}_{e}$ are the effective elasticity tensors that describe the myocytes and the extracellular matrix. These tensors can be obtained by carrying out a homogenization process at the finer hierarchical level, i.e. at the porescale shown in Fig. 1. These tensors ${\mathbb {C}}_{i}$ and ${\mathbb {C}}_{e}$ are the effective elasticity tensors obtained in (Burridge and Keller (1981); Penta et al. (2020)) for a standard poroelastic material. These effective elasticity tensors possess major and minor symmetries as proved in Mei and Vernescu (2010). This means we can write the fourth rank effective elasticity tensors in components as ${\mathcal {C}}_{pqrs}^{i}$ and ${\mathcal {C}}_{pqrs}^{e}$, for $p, q, r, s=1, 2, 3$. Therefore, we have

7a $$\begin{aligned}&{\mathcal {C}}_{pqrs}^{i}={\mathcal {C}}_{qprs}^{i}={\mathcal {C}}_{pqsr}^{i}={\mathcal {C}}_{rspq}^{i}, \end{aligned}$$

7b $$\begin{aligned}&{\mathcal {C}}_{pqrs}^{e}={\mathcal {C}}_{qprs}^{e}={\mathcal {C}}_{pqsr}^{e}={\mathcal {C}}_{rspq}^{e}. \end{aligned}$$

The second rank tensors $\varvec{\alpha }_{i}$ and $\varvec{\alpha }_{e}$ appearing in (5a) and (5b) are the effective Biot’s tensors of coefficients in the myocyte and the extracellular matrix, respectively. These tensors also have been obtained from the homogenization at finer hierarchical scales. The second rank tensors ${\varvec{\alpha }}_{i}$ and ${\varvec{\alpha }}_{e}$ are related to the ratio of fluid to solid volume changes at constant pressure in their respective poroelastic phases.

In the myocytes and the extracellular matrix, we also have a Darcy’s law governing the fluid flow. That is,

8a $$\begin{aligned}&\textbf{w}_{i}=-{{\textsf{K}}}_{i}\nabla p_{i}\qquad \text{ in } \quad \Omega _{i}, \end{aligned}$$

8b $$\begin{aligned}&\textbf{w}_{e}=-{{\textsf{K}}}_{e}\nabla p_{e}\qquad \text{ in } \quad \Omega _{e} ,\end{aligned}$$

where ${{\textsf{K}}}_{i}$ and ${{\textsf{K}}}_{e}$ are the hydraulic conductivities in the myocyte and extracellular matrix, respectively, and the $\textbf{w}_{i}$ and $\textbf{w}_{e}$ are the relative fluid–solid velocities in the myocytes and extracellular matrix, respectively.

The last governing equation of each compartment is the conservation of mass equation given by

9a $$\begin{aligned}&\frac{{\dot{p}}_{i}}{{M}_{i}}=-{\varvec{\alpha }}_{i}:\zeta ( \dot{\textbf{u}}_{i})-\nabla \cdot \textbf{w}_{i} \qquad \text{ in } \quad \Omega _{i}, \end{aligned}$$

9b $$\begin{aligned}&\frac{{\dot{p}}_{e}}{{M}_{e}}=-{\varvec{\alpha }}_{e}:\zeta (\dot{\textbf{u}}_{e})-\nabla \cdot \textbf{w}_{e}\qquad \text{ in } \quad \Omega _{e}, \end{aligned}$$

in the myocyte and extracellular matrix. We have the Biot’s moduli ${M}_{i}$ and ${M}_{e}$ in each compartment, which are also coefficients that can be obtained from the homogenization process at finer hierarchical scales. These moduli ${M}_{i}$ and ${M}_{e}$ can be described physically as poroelastic coefficients that depend on the porescale geometry, porosity and the fluid bulk modulus. They also depend on the elastic properties of the myocyte and matrix, respectively. These coefficients ${M}_{i}$ and ${M}_{e}$ can be interpreted as the inverse of the variation of fluid volume in response to a variation in pore pressure. The have been proved to be positive definite in Mei and Vernescu (2010).

Finally we need to close the problem by prescribing conditions on the interface $\Gamma$. These are continuity of electric current densities, jump in electric potentials, continuity of stress, continuity of elastic displacements and the continuity of fluxes and pressures between the myocytes and the extracellular matrix

10a $$\begin{aligned}&{\textsf{G}}_{i}\nabla \phi _i\cdot \textbf{n}={\textsf{G}}_{e}\nabla \phi _e\cdot \textbf{n}\qquad \textrm{on}\quad \Gamma , \end{aligned}$$

10b $$\begin{aligned}&\phi _i-\phi _e={V}\qquad \textrm{on}\quad \Gamma , \end{aligned}$$

10c $$\begin{aligned}&{\textsf{T}}_i\cdot \textbf{n}={\textsf{T}}_e\cdot \textbf{n}\qquad \textrm{on}\quad \Gamma , \end{aligned}$$

10d $$\begin{aligned}&\textbf{u}_i=\textbf{u}_e\qquad \textrm{on}\quad \Gamma , \end{aligned}$$

10e $$\begin{aligned}&\textbf{w}_e\cdot \textbf{n}={\textbf{w}}_i \cdot \textbf{n}\qquad \textrm{on}\quad \Gamma , \end{aligned}$$

10f $$\begin{aligned}&p_i=p_e\qquad \textrm{on}\quad \Gamma , \end{aligned}$$

where *V* is a given and is the potential drop across the membrane (Richardson and Chapman 2011) and $\textbf{n}$ is the normal to the interface $\Gamma$ pointing into the myocyte.

The problem is also to be closed by appropriate boundary conditions on the external boundary $\partial \Omega$. The latter could be, for example, of Dirichlet–Neumann type, as noted in Ramírez-Torres et al. (2018). The conditions on the external boundary typically do not play a role in the derivation of results carried out by formal asymptotic homogenization.

Now that we have set up the problem, we are able perform a multiscale analysis in order to derive the new model. To do this, we will (a) non-dimensionalize the problem we have set up in this section, (b) introduce two well-separated length scales, (c) apply the asymptotic homogenization technique to the non-dimensionalized problem, and (d) derive the novel effective governing equations for the myocardium.

## Multiscale analysis

To progress with our analysis, we determine that there exists two different length scales in the myocardium. We denote the average size of the whole heart by *L* and this is the macroscale. The scale at which we clearly see the myocytes resolved from the surrounding matrix is denoted by *d*, the microscale. So as to correctly capture the behaviour of the material, it is important to emphasize the difference between such scales; therefore, we carry out a non-dimensional analysis of the equations described in Sect. 2.

### Non-dimensionalization of the problem

In order to derive our model, we must begin with equations that are in non-dimensional form as this allows us to clearly understand the contribution of each of the relevant fields. Since the model is describing the myocardium, it is best to derive in a general form so that it can later be specified to certain conditions or diseases. This means that we perform a formal non-dimensionalization that will highlight the proper asymptotic behaviour of each of the relevant fields instead of one motivated by heart/disease-specific parameters. We choose to scale the spatial variable and the elastic displacement as well as the stresses and elasticity tensors, by the characteristic length scale *L* of the domain. We also assume that the system is characterized by a reference pressure gradient *C*, and that the characteristic fluid velocity is given by the typical parabolic profile proportional to that of a Newtonian fluid slowly flowing in a cylinder of radius *d*. We have that $\Phi _0$ is the typical potential drop and ${\textsf{G}}_0$ is the typical conductance. We therefore non-dimensionalize by assuming the following

11 $$\begin{aligned} \begin{aligned} {\textbf{x}}&=L \mathbf {x'},\quad {\mathbb {C}}_{i}=CL{\mathbb {C}}_{i}',\quad {\mathbb {C}}_{e}=CL{\mathbb {C}}_{e}', \quad {\textsf{T}}_i=CL {\textsf{T}}_i', \\ {\textsf{T}}_e&=CL{\textsf{T}}_e',\quad \textbf{u}_e=L\textbf{u}'_e, \quad \textbf{u}_i=L\textbf{u}'_i,\\&\quad \phi _i=\Phi _0\phi _i',\quad \phi _e=\Phi _0\phi _e', \\ V&=\Phi _0V',\quad {\textsf{G}}_i={\textsf{G}}_0 {\textsf{G}}_i',\quad {\textsf{G}}_e={\textsf{G}}_0 {\textsf{G}}_e', \, \textbf{B}=\frac{L}{{\textsf{G}}_0\Phi _0} \textbf{B}',\\ \textbf{v}&=\frac{Cd^2}{\mu } \textbf{v}',\quad p_i=CLp_i', \quad p_e=CLp_e'. \end{aligned} \end{aligned}$$

We are then able to use (11) and note that

12 $$\begin{aligned} \nabla =\frac{1}{L}\nabla ' \end{aligned}$$

We now substitute each of these into (1a)–(3b), (5a)–(5b), (8a)–(9b), and interface conditions (10a)–(10f) obtain the following non-dimensional form of the system of PDEs where we have dropped the “primes” for the sake of simplifying the notation

13a $$\begin{aligned}&\nabla \cdot ({\textsf{G}}_{i}\nabla \phi _i)={\hat{\beta }} (\phi _i-\phi _e) \qquad \textrm{in}\quad \Omega _i, \end{aligned}$$

13b $$\begin{aligned}&\nabla \cdot ({\textsf{G}}_{e}\nabla \phi _e)=-{\hat{\beta }} (\phi _i-\phi _e) \qquad \textrm{in}\quad \Omega _e, \end{aligned}$$

13c $$\begin{aligned}&\textbf{j}_i=-{\textsf{G}}_{i}\nabla \phi _i \qquad \textrm{in}\quad \Omega _i, \end{aligned}$$

13d $$\begin{aligned}&\textbf{j}_e=-{\textsf{G}}_{e}\nabla \phi _e \qquad \textrm{in}\quad \Omega _e, \end{aligned}$$

13e $$\begin{aligned}&\nabla \cdot {\textsf{T}}_i= -{{\textsf{G}}_i\nabla \phi _i\times \textbf{B}}\qquad \textrm{in}\quad \Omega _i, \end{aligned}$$

13f $$\begin{aligned}&\nabla \cdot {\textsf{T}}_e= -{{\textsf{G}}_e\nabla \phi _e\times \textbf{B}}\qquad \textrm{in}\quad \Omega _e, \end{aligned}$$

13g $$\begin{aligned}&{{\textsf{T}}}_{i}={\mathbb {C}}_{i}:\zeta (\textbf{u}_{i})-{\varvec{\alpha }}_{i}p_{i} \qquad \text{ in } \quad \Omega _{i}, \end{aligned}$$

13h $$\begin{aligned}&{{\textsf{T}}}_{e}={\mathbb {C}}_{e}:\zeta ( \textbf{u}_{e})-{\varvec{\alpha }}_{e}p_{e} \qquad \text{ in } \quad \Omega _{e}, \end{aligned}$$

13i $$\begin{aligned}&\textbf{w}_{i}=-{{\textsf{K}}}_{i}\nabla p_{i}\qquad \text{ in } \quad \Omega _{i}, \end{aligned}$$

13j $$\begin{aligned}&\textbf{w}_{e}=-{{\textsf{K}}}_{e}\nabla p_{e}\qquad \text{ in } \quad \Omega _{e} \end{aligned}$$

13k $$\begin{aligned}&\frac{{\dot{p}}_{i}}{{M}_{i}}=-{\varvec{\alpha }}_{i}:\zeta (\dot{\textbf{u}}_{i})-\nabla \cdot \textbf{w}_{i} \qquad \text{ in } \quad \Omega _{i}, \end{aligned}$$

13l $$\begin{aligned}&\frac{{\dot{p}}_{e}}{{M}_{e}}=-{\varvec{\alpha }}_{e}:\zeta (\dot{\textbf{u}}_{e})-\nabla \cdot \textbf{w}_{e}\qquad \text{ in } \quad \Omega _{e}, \end{aligned}$$

13m $$\begin{aligned}&{\textsf{G}}_{i}\nabla \phi _i\cdot \textbf{n}={\textsf{G}}_{e}\nabla \phi _e\cdot \textbf{n}\qquad \textrm{on}\quad \Gamma , \end{aligned}$$

13n $$\begin{aligned}&\phi _i-\phi _e={V}\qquad \textrm{on}\quad \Gamma , \end{aligned}$$

13o $$\begin{aligned}&{\textsf{T}}_i\cdot \textbf{n}={\textsf{T}}_e\cdot \textbf{n}\qquad \textrm{on}\quad \Gamma , \end{aligned}$$

13p $$\begin{aligned}&\textbf{u}_i=\textbf{u}_e\qquad \textrm{on}\quad \Gamma , \end{aligned}$$

13q $$\begin{aligned}&\textbf{w}_e\cdot \textbf{n}={\textbf{w}}_i \cdot \textbf{n}\qquad \textrm{on}\quad \Gamma , \end{aligned}$$

13r $$\begin{aligned}&p_i=p_e\qquad \textrm{on}\quad \Gamma , \end{aligned}$$

where we can write the following dimensionless parameter

14 $$\begin{aligned} {\hat{\beta }}=\frac{\beta G L^2}{G_0}. \end{aligned}$$

Now that we have our problem in non-dimensional form, we are ready to apply the asymptotic homogenization technique which we will use to upscale the PDEs (13a–13r) by making the formal assumption that the microscale and the macroscale are well separated. We follow the technique as outlined in Miller and Penta (2023, 2021a) with the assumptions of microscale periodicity and macroscopic uniformity and summarized in Appendix A.1 with associated remarked assumptions.

### Multiple scales expansion

We apply the assumptions of the asymptotic homogenization technique (92) and (93) to Eqs. (13a–13r). We then obtain, accounting for the periodicity, the following

15a $$\begin{aligned}&\epsilon ^2\nabla _\textbf{x}\cdot ({\textsf{G}}_{i}\nabla _\textbf{x}\phi _i^\epsilon )+\epsilon \nabla _\textbf{x}\cdot ({\textsf{G}}_{i}\nabla _\textbf{y}\phi _i^\epsilon )+\epsilon \nabla _\textbf{y}\cdot ({\textsf{G}}_{i}\nabla _\textbf{x}\phi _i^\epsilon )\nonumber \\&\quad +\nabla _\textbf{y}\cdot ({\textsf{G}}_{i}\nabla _\textbf{y}\phi _i^\epsilon )=\epsilon ^2{\hat{\beta }} (\phi _i^\epsilon -\phi _e^\epsilon )~~ \text{ in } ~ \Omega _{i} \end{aligned}$$

15b $$\begin{aligned}&\epsilon ^2\nabla _\textbf{x}\cdot ({\textsf{G}}_{e}\nabla _\textbf{x}\phi _e^\epsilon )+\epsilon \nabla _\textbf{x}\cdot ({\textsf{G}}_{e}\nabla _\textbf{y}\phi _e^\epsilon )+\epsilon \nabla _\textbf{y}\cdot ({\textsf{G}}_{e}\nabla _\textbf{x}\phi _e^\epsilon ))\nonumber \\&\quad +\nabla _\textbf{y}\cdot ({\textsf{G}}_{e}\nabla _\textbf{y}\phi _e^\epsilon =-\epsilon ^2{\hat{\beta }} (\phi _i^\epsilon -\phi _e^\epsilon )~~ \text{ in } ~ \Omega _{e} \end{aligned}$$

15c $$\begin{aligned}&\epsilon \textbf{j}_i^\epsilon =-\epsilon {\textsf{G}}_{i}\nabla _\textbf{x}\phi _i^\epsilon -{\textsf{G}}_{i}\nabla _\textbf{y}\phi _i^\epsilon ~~\text{ in } ~\Omega _{i} \end{aligned}$$

15d $$\begin{aligned} \epsilon \textbf{j}_e^\epsilon =-\epsilon {\textsf{G}}_{e}\nabla _\textbf{x}\phi _e^\epsilon \nonumber -{\textsf{G}}_{e}\nabla _\textbf{y}\phi _e^\epsilon ~~ \text{ in } ~ \Omega _{e} \end{aligned}$$

15e $$\begin{aligned}&\epsilon \nabla _\textbf{x}\cdot {\textsf{T}}_i^\epsilon +\nabla _\textbf{y}\cdot {\textsf{T}}_i^\epsilon = -{\epsilon {\textsf{G}}_i\nabla _\textbf{x}\phi _i^\epsilon \times \textbf{B}^\epsilon }\nonumber \\&\quad -{{\textsf{G}}_i\nabla _\textbf{y}\phi _i^\epsilon \times \textbf{B}^\epsilon }~~ \text{ in } ~ \Omega _{i} \end{aligned}$$

15f $$\begin{aligned}&\epsilon \nabla _\textbf{x}\cdot {\textsf{T}}_e^\epsilon +\nabla _\textbf{y}\cdot {\textsf{T}}_e^\epsilon = -{\epsilon {\textsf{G}}_e\nabla _\textbf{x}\phi _e^\epsilon \times \textbf{B}^\epsilon }\nonumber \\&\quad -{{\textsf{G}}_e\nabla _\textbf{y}\phi _e^\epsilon \times \textbf{B}^\epsilon }~~\text{ in } ~ \Omega _{e} \end{aligned}$$

15g $$\begin{aligned}&\epsilon {{\textsf{T}}}_{i}^\epsilon ={\mathbb {C}}_{i}:\zeta _y (\textbf{u}_{i}^\epsilon )+\epsilon {\mathbb {C}}_{i}:\zeta _x (\textbf{u}_{i}^\epsilon )-\epsilon {\varvec{\alpha }}_{i}p_{i}^\epsilon\quad \text{ in } ~\Omega _{e} \end{aligned}$$

15h $$\begin{aligned}&\epsilon {{\textsf{T}}}_{e}^\epsilon ={\mathbb {C}}_{e}:\zeta _y (\textbf{u}_{e}^\epsilon )+\epsilon {\mathbb {C}}_{e}:\zeta _x( \textbf{u}_{e}^\epsilon )-\epsilon {\varvec{\alpha }}_{e}p_{e}^\epsilon\quad \text{ in } ~\Omega _{e} \end{aligned}$$

15i $$\begin{aligned}&\epsilon \textbf{w}_{i}^\epsilon =-{{\textsf{K}}}_{i}\nabla _y p_{i}^\epsilon -\epsilon {{\textsf{K}}}_{i}\nabla _x p_{i}^\epsilon\quad \text{ in } ~ \Omega _{i} \end{aligned}$$

15j $$\begin{aligned}&\epsilon \textbf{w}_{e}^\epsilon =-{{\textsf{K}}}_{e}\nabla _y p_{e}^\epsilon -\epsilon {{\textsf{K}}}_{e}\nabla _x p_{e}^\epsilon ~~ \text{ in } ~\Omega _{e} \end{aligned}$$

15k $$\begin{aligned}&\epsilon \frac{{\dot{p}}_{i}^\epsilon }{{M}_{i}}=-{\varvec{\alpha }}_{i}:\zeta _y ( \dot{\textbf{u}}_{i}^\epsilon )-\epsilon {\varvec{\alpha }}_{i}:\zeta _x (\dot{\textbf{u}}_{i}^\epsilon )\nonumber \\&\quad -\nabla _y\cdot \textbf{w}_{i}^\epsilon -\epsilon \nabla _x\cdot \textbf{w}_{i}^\epsilon ~~ \text{ in } ~ \Omega _{i} \end{aligned}$$

15l $$\begin{aligned}&\epsilon \frac{{\dot{p}}_{e}^\epsilon }{{M}_{e}}=-{\varvec{\alpha }}_{e}:\zeta _y (\dot{\textbf{u}}_{e}^\epsilon )-\epsilon {\varvec{\alpha }}_{e}:\zeta _x (\dot{\textbf{u}}_{e}^\epsilon )\nonumber \\&\quad -\nabla _y\cdot \textbf{w}_{e}^\epsilon -\epsilon \nabla _x\cdot \textbf{w}_{e}^\epsilon\quad \text{ in } ~\Omega _{\textrm{e}} \end{aligned}$$

15m $$\begin{aligned}&{\textsf{G}}_{i}\nabla _\textbf{y}\phi _i^\epsilon \cdot \textbf{n}+\epsilon {\textsf{G}}_{i}\nabla _\textbf{x}\phi _i^\epsilon \cdot \textbf{n}\nonumber \\&\quad ={\textsf{G}}_{e}\nabla _\textbf{y}\phi _e^\epsilon \cdot \textbf{n}+\epsilon {\textsf{G}}_{e}\nabla _\textbf{x}\phi _e^\epsilon \cdot \textbf{n},&~~ \text{ on } ~\Gamma \end{aligned}$$

15n $$\begin{aligned}&\phi _i^\epsilon -\phi _e^\epsilon ={V^\epsilon }&~~ \text{ on } ~\Gamma , \end{aligned}$$

15o $$\begin{aligned}&{{\textsf{T}}}_{i}^\epsilon \textbf{n}={{\textsf{T}}}_{e}^\epsilon \textbf{n}&~~ \text{ on } ~ \Gamma , \end{aligned}$$

15p $$\begin{aligned}&\textbf{u}_{i}^\epsilon =\textbf{u}_{e}^\epsilon ~~ \text{ on } ~\Gamma \end{aligned}$$

15q $$\begin{aligned}&\textbf{w}_{i}^\epsilon \cdot \textbf{n}=\textbf{w}_{e}^\epsilon \cdot \textbf{n}&~~ \text{ on }~\Gamma \end{aligned}$$

15r $$\begin{aligned}&p_{i}^\epsilon =p_{e}^\epsilon ~~\text{ on }~ \Gamma \end{aligned}$$

Now that we have the expansion, we can substitute power series of the type (93) into the relevant fields in (15a–15r). This allows us to equate the coefficients of $\epsilon ^l$ for $l=0,1,\ldots ,$ and therefore, we derive the macroscale model for the material in terms of the relevant leading-order fields.

If a field in the asymptotic expansion retains a microscale dependence, we apply the integral average. This can be defined as

16 $$\begin{aligned} \langle \varphi \rangle _k=\frac{1}{|\Omega |}\int _{\Omega _i}\varphi (\textbf{x,y},t)d\textbf{y} \quad k=i,e, \end{aligned}$$

where due to $\textbf{y}$-periodicity the integral average is taken over one representative cell and $|\Omega |$ is the volume of the domain, where $|\Omega |=|\Omega _i|+|\Omega _e|$. Due to $\textbf{y}$-periodicity, the integral average is over one representative cell, and therefore, (16) represents a cell average.

We are now ready to consider the coefficients of different powers of $\epsilon$ in the multiple scales expansion. Equating first the coefficients of $\epsilon ^0$

17a $$\begin{aligned}&\nabla _\textbf{y}\cdot ({\textsf{G}}_{i}\nabla _\textbf{y}\phi _i^{(0)})=0,\qquad \text{ in }\quad \Omega _i \end{aligned}$$

17b $$\begin{aligned}&\nabla _\textbf{y}\cdot ({\textsf{G}}_{e}\nabla _\textbf{y}\phi _e^{(0)})=0,\qquad \text{ in }\quad \Omega _e \end{aligned}$$

17c $$\begin{aligned}&{\textsf{G}}_{i}\nabla _\textbf{y}\phi _i^{(0)}=0,\qquad \text{ in }\quad \Omega _i \end{aligned}$$

17d $$\begin{aligned}&{\textsf{G}}_{e}\nabla _\textbf{y}\phi _e^{(0)}=0,\qquad \text{ in }\quad \Omega _e \end{aligned}$$

17e $$\begin{aligned}&\nabla _\textbf{y}\cdot {\textsf{T}}_i^{(0)}=-{{\textsf{G}}_i\nabla _\textbf{y}\phi _i^{(0)}\times \textbf{B}^{(0)}},\qquad \text{ in }\quad \Omega _i \end{aligned}$$

17f $$\begin{aligned}&\nabla _\textbf{y}\cdot {\textsf{T}}_e^{(0)}=-{{\textsf{G}}_e\nabla _\textbf{y}\phi _e^{(0)}\times \textbf{B}^{(0)}},\qquad \text{ in }\quad \Omega _e \end{aligned}$$

17g $$\begin{aligned}&{\mathbb {C}}_{i}:\zeta _y (\textbf{u}_{i}^{(0)})=0,\qquad \text{ in }\quad \Omega _i \end{aligned}$$

17h $$\begin{aligned}&{\mathbb {C}}_{e}:\zeta _y (\textbf{u}_{e}^{(0)})=0,\qquad \text{ in }\quad \Omega _e \end{aligned}$$

17i $$\begin{aligned}&{{\textsf{K}}}_{i}\nabla _y p_{i}^{(0)}=0,\qquad \text{ in }\quad \Omega _i \end{aligned}$$

17j $$\begin{aligned}&{{\textsf{K}}}_{e}\nabla _y p_{e}^{(0)}=0,\qquad \text{ in }\quad \Omega _e \end{aligned}$$

17k $$\begin{aligned}&{\varvec{\alpha }}_{i}:\zeta _y (\dot{\textbf{u}}_{i}^{(0)})+\nabla _y\cdot \textbf{w}_{i}^{(0)}=0,\qquad \text{ in }\quad \Omega _i \end{aligned}$$

17l $$\begin{aligned}&{\varvec{\alpha }}_{e}:\zeta _y (\dot{\textbf{u}}_{e}^{(0)})+\nabla _y\cdot \textbf{w}_{e}^{(0)}=0,\qquad \text{ in }\quad \Omega _e \end{aligned}$$

17m $$\begin{aligned}&{\textsf{G}}_{i}\nabla _\textbf{y}\phi _i^{(0)}\cdot \textbf{n}={\textsf{G}}_{e}\nabla _\textbf{y}\phi _e^{(0)}\cdot \textbf{n},\qquad \text{ on }\quad \Gamma \end{aligned}$$

17n $$\begin{aligned}&\phi _i^{(0)}-\phi _e^{(0)}={V^{(0)}},\qquad \text{ on }\quad \Gamma \end{aligned}$$

17o $$\begin{aligned}&{{\textsf{T}}}_{i}^{(0)} \textbf{n}={{\textsf{T}}}_{e}^{(0)} \textbf{n},\qquad \text{ on }\quad \Gamma \end{aligned}$$

17p $$\begin{aligned}&\textbf{u}_{i}^{(0)}=\textbf{u}_{e}^{(0)},\qquad \text{ on }\quad \Gamma \end{aligned}$$

17q $$\begin{aligned}&\textbf{w}_{i}^{(0)}\cdot \textbf{n}=\textbf{w}_{e}^{(0)}\cdot \textbf{n},\qquad \text{ on }\quad \Gamma \end{aligned}$$

17r $$\begin{aligned}&p_{i}^{(0)}=p_{e}^{(0)}.\qquad \text{ on }\quad \Gamma \end{aligned}$$

Equations (17g) and (17h) show that $\textbf{u}_i^{(0)}$ and $\textbf{u}_e^{(0)}$ are rigid body motions, and since we have $\textbf{y}$-periodicity, the leading-order elastic displacements in each domain do not depend on the microscale variable $\textbf{y}$. That is,

18 $$\begin{aligned} \textbf{u}_i^{(0)}=\textbf{u}_i^{(0)}(\textbf{x},t)\quad \text{ and }\quad \textbf{u}_e^{(0)}=\textbf{u}_e^{(0)}(\textbf{x},t). \end{aligned}$$

Due to the interface condition (17p) $\textbf{u}_i^{(0)}=\textbf{u}_e^{(0)}$ on $\Gamma$, we will use that

19 $$\begin{aligned} \textbf{u}^{(0)}=\textbf{u}_i^{(0)}=\textbf{u}_e^{(0)} \end{aligned}$$

in the remainder of this work.

We also see from (17i) and (17j) with interface condition (17r) that the leading-order pressures $p_i^{(0)}$ and $p_e^{(0)}$ do not depend on the microscale variable. That is,

20 $$\begin{aligned} p^{(0)}=p_i^{(0)}(\textbf{x}, t)=p_e^{(0)}(\textbf{x}, t). \end{aligned}$$

We chose that the difference in electric potentials $V^{(0)}$ is given and does not depend on the microscale variable $\textbf{y}$, see (94). We are able to write the $\epsilon ^0$ problem for ${\phi _i}^{(0)}$ and ${\phi _e}^{(0)}$. To do this, we define a new variable,

21 $$\begin{aligned} \bar{\phi }_e^{(0)}=\phi _e^{(0)}+V^{(0)}. \end{aligned}$$

This allows us to write the $\epsilon ^0$ problem in terms of ${\phi _i}^{(0)}$ and the new variable $\bar{\phi }_e^{(0)}$

22a $$\begin{aligned}&\nabla _\textbf{y}\cdot ({\textsf{G}}_{i}\nabla _\textbf{y}\phi _i^{(0)})=0,\quad \text{ in }\quad \Omega _i \end{aligned}$$

22b $$\begin{aligned}&\nabla _\textbf{y}\cdot ({\textsf{G}}_{e}\nabla _\textbf{y}\bar{\phi }_e^{(0)})=0,\quad \text{ in }\quad \Omega _e \end{aligned}$$

22c $$\begin{aligned}&\phi _i^{(0)}=\bar{\phi }_e^{(0)},\quad \text{ on }\quad \Gamma \end{aligned}$$

22d $$\begin{aligned}&{\textsf{G}}_{i}\nabla _\textbf{y}\phi _i^{(0)}\cdot \textbf{n}={\textsf{G}}_{e}\nabla _\textbf{y}\bar{\phi }_e^{(0)}\cdot \textbf{n}.\quad \text{ on }\quad \Gamma \end{aligned}$$

The problem (22a)–(22d) is of linear elastic type. The problem has the jump condition (22d) between the current densities and the continuity of the zeroth-order electric potentials (22c). Problems of this type have been studied in the literature (Bakhvalov and Panasenko 2012; Cioranescu and Donato 1999) where it has been proved that the only solutions to these problems are constant with respect to the microscale variable $\textbf{y}$. Therefore, (22a)–(22d) gives the solution that ${\phi _i}^{(0)}$ and ${\bar{\phi }_e}^{(0)}$ do not depend on the microscale. This means that it follows that both ${\phi _i}^{(0)}$ and ${{\phi }_e}^{(0)}$ do not depend on that microscale. Therefore, we can write

23a $$\begin{aligned} {\phi _i}^{(0)}&={\phi _i}^{(0)}(\textbf{x},t) \end{aligned}$$

23b $$\begin{aligned} {\phi _e}^{(0)}&={\phi _e}^{(0)}(\textbf{x},t). \end{aligned}$$

Therefore, the balance Eqs. (17e) and (17f) will reduce to

24a $$\begin{aligned}&\nabla _\textbf{y}\cdot {\textsf{T}}_i^{(0)}=0\quad \text{ in }\quad \Omega _i, \end{aligned}$$

24b $$\begin{aligned}&\nabla _\textbf{y}\cdot {\textsf{T}}_e^{(0)}=0\quad \text{ in }\quad \Omega _e. \end{aligned}$$

We now equate the coefficients of $\epsilon ^1$

25a $$\begin{aligned}&\nabla _\textbf{y}\cdot ({\textsf{G}}_{i}\nabla _\textbf{x}\phi _i^{(0)})+\nabla _\textbf{y}\cdot ({\textsf{G}}_{i}\nabla _\textbf{y}\phi _i^{(1)})=0\quad \text{ in }~~\Omega _i , \end{aligned}$$

25b $$\begin{aligned}&\nabla _\textbf{y}\cdot ({\textsf{G}}_{e}\nabla _\textbf{x}\phi _e^{(0)})+\nabla _\textbf{y}\cdot ({\textsf{G}}_{e}\nabla _\textbf{y}\phi _e^{(1)})=0\quad \text{ in }~~\Omega _e , \end{aligned}$$

25c $$\begin{aligned}&\textbf{j}_i^{(0)}=-{\textsf{G}}_{i}\nabla _\textbf{x}\phi _i^{(0)} -{\textsf{G}}_{i}\nabla _\textbf{y}\phi _i^{(1)}\quad \text{ in }~~\Omega _i , \end{aligned}$$

25d $$\begin{aligned}&\textbf{j}_e^{(0)}=-{\textsf{G}}_{e}\nabla _\textbf{x}\phi _e^{(0)} -{\textsf{G}}_{e}\nabla _\textbf{y}\phi _e^{(1)}\quad \text{ in }~~\Omega _e , \end{aligned}$$

25e $$\begin{aligned}&\nabla _\textbf{x}\cdot {\textsf{T}}_i^{(0)}+\nabla _\textbf{y}\cdot {\textsf{T}}_i^{(1)}= -{{\textsf{G}}_i\nabla _\textbf{x}\phi _i^{(0)}\times \textbf{B}^{(0)}}\nonumber \\&\quad -{{\textsf{G}}_i\nabla _\textbf{y}\phi _i^{(1)}\times \textbf{B}^{(0)}}\quad \text{ in }~~\Omega _i , \end{aligned}$$

25f $$\begin{aligned}&\nabla _\textbf{x}\cdot {\textsf{T}}_e^{(0)}+\nabla _\textbf{y}\cdot {\textsf{T}}_e^{(1)}= -{{\textsf{G}}_e\nabla _\textbf{x}\phi _e^{(0)}\times \textbf{B}^{(0)}}\nonumber \\&\quad -{{\textsf{G}}_e\nabla _\textbf{y}\phi _e^{(1)}\times \textbf{B}^{(0)}}\quad \text{ in }~~\Omega _e , \end{aligned}$$

25g $$\begin{aligned}&{{\textsf{T}}}_{i}^{(0)}={\mathbb {C}}_{i}:\zeta _y (\textbf{u}_{i}^{(1)})+{\mathbb {C}}_{i}:\zeta _x (\textbf{u}_{i}^{(0)})\nonumber \\&\quad -{\varvec{\alpha }}_{i}p_{i}^{(0)}\quad \text{ in }~~\Omega _i , \end{aligned}$$

25h $$\begin{aligned}&{{\textsf{T}}}_{e}^{(0)}={\mathbb {C}}_{e}:\zeta _y (\textbf{u}_{e}^{(1)})+{\mathbb {C}}_{e}:\zeta _x( \textbf{u}_{e}^{(0)})\nonumber \\&\quad -{\varvec{\alpha }}_{e}p_{e}^{(0)}\quad \text{ in }~~\Omega _e , \end{aligned}$$

25i $$\begin{aligned}&\textbf{w}_{i}^{(0)}=-{{\textsf{K}}}_{i}\nabla _y p_{i}^{(1)}-{{\textsf{K}}}_{i}\nabla _x p_{i}^{(0)}\quad \text{ in }~~\Omega _i , \end{aligned}$$

25j $$\begin{aligned}&\textbf{w}_{e}^{(0)}=-{{\textsf{K}}}_{e}\nabla _y p_{e}^{(1)}-{{\textsf{K}}}_{e}\nabla _x p_{e}^{(0)}\quad \text{ in }~~\Omega _e , \end{aligned}$$

25k $$\begin{aligned}&\frac{{\dot{p}}_{i}^{(0)}}{{M}_{i}}=-{\varvec{\alpha }}_{i}:\zeta _y (\dot{\textbf{u}}_{i}^{(1)})-{\varvec{\alpha }}_{i}:\zeta _x ( \dot{\textbf{u}}_{i}^{(0)})-\nabla _y\cdot \textbf{w}_{i}^{(1)}\nonumber \\&\quad -\nabla _x\cdot \textbf{w}_{i}^{(0)}\quad \text{ in }~~\Omega _i , \end{aligned}$$

25l $$\begin{aligned}&\frac{{\dot{p}}_{e}^{(0)}}{{M}_{e}}=-{\varvec{\alpha }}_{e}:\zeta _y (\dot{\textbf{u}}_{e}^{(1)})-{\varvec{\alpha }}_{e}:\zeta _x ( \dot{\textbf{u}}_{e}^{(0)})-\nabla _y\cdot \textbf{w}_{e}^{(1)}\nonumber \\&\quad -\nabla _x\cdot \textbf{w}_{e}^{(0)}\quad \text{ in }~~\Omega _e, \end{aligned}$$

25m $$\begin{aligned}&{\textsf{G}}_{i}\nabla _\textbf{y}\phi _i^{(1)}\cdot \textbf{n}+{\textsf{G}}_{i}\nabla _\textbf{x}\phi _i^{(0)}\cdot \textbf{n}\nonumber \\&\quad ={\textsf{G}}_{e}\nabla _\textbf{y}\phi _e^{(1)}\cdot \textbf{n}+{\textsf{G}}_{e}\nabla _\textbf{x}\phi _e^{(0)}\cdot \textbf{n}\quad \text{ on }~~\Gamma , \end{aligned}$$

25n $$\begin{aligned}&\phi _i^{(1)}-\phi _e^{(1)}={V^{(1)}}\quad \text{ on }~~\Gamma , \end{aligned}$$

25o $$\begin{aligned}&{{\textsf{T}}}_{i}^{(1)} \textbf{n}={{\textsf{T}}}_{e}^{(1)} \textbf{n}\quad \text{ on }~~\Gamma , \end{aligned}$$

25p $$\begin{aligned}&\textbf{u}_{i}^{(1)}=\textbf{u}_{e}^{(1)}\quad \text{ on }~~\Gamma , \end{aligned}$$

25q $$\begin{aligned}&\textbf{w}_{i}^{(1)}\cdot \textbf{n}=\textbf{w}_{e}^{(1)}\cdot \textbf{n}\quad \text{ on }~~\Gamma , \end{aligned}$$

25r $$\begin{aligned}&p_{i}^{(1)}=p_{e}^{(1)}\quad \text{ on }~~\Gamma . \end{aligned}$$

With the coefficients of $\epsilon ^0$ and $\epsilon ^{1},$ we can use these equations to form problems for the order 1 electric potentials, elastic displacements and the relative fluid–solid velocity. This will allow us to understand the electrostatic and poroelastic behaviours of the myocardium.

### Problem for electric potentials $\phi _i^{(1)}$ and $\phi _e^{(1)}$

Using the balance Eqs. (25a), (25b), with the interface conditions (25m) and (25o) we form the following steady-state problem for the electric potentials $\phi _i^{(1)}$ and $\phi _e^{(1)}$, i.e. the electrostatic problem. We have

26a $$\begin{aligned}&\nabla _\textbf{y}\cdot ({\textsf{G}}_{i}\nabla _\textbf{x}\phi _i^{(0)})+\nabla _\textbf{y}\cdot ({\textsf{G}}_{i}\nabla _\textbf{y}\phi _i^{(1)})=0\qquad \text{ in }\quad \Omega _i, \end{aligned}$$

26b $$\begin{aligned}&\nabla _\textbf{y}\cdot ({\textsf{G}}_{e}\nabla _\textbf{x}\phi _e^{(0)})+\nabla _\textbf{y}\cdot ({\textsf{G}}_{e}\nabla _\textbf{y}\phi _e^{(1)})=0\qquad \text{ in }\quad \Omega _e, \end{aligned}$$

26c $$\begin{aligned}&\phi _i^{(1)}-\phi _e^{(1)}={V^{(1)}}\qquad \text{ on }\quad \Gamma , \end{aligned}$$

26d $$\begin{aligned}&({\textsf{G}}_{i}\nabla _\textbf{y}\phi _i^{(1)}-{\textsf{G}}_{e}\nabla _\textbf{y}\phi _e^{(1)})\cdot \textbf{n}\nonumber \\&\quad =({\textsf{G}}_{e}\nabla _\textbf{x}\phi _e^{(0)}-{\textsf{G}}_{i}\nabla _\textbf{x}\phi _i^{(0)})\cdot \textbf{n}\qquad \text{ on }\quad \Gamma . \end{aligned}$$

The problem (26a)–(26d) is linear, and therefore, we can propose the following ansatz

27a $$\begin{aligned}&\phi _i^{(1)}=\Phi _i\nabla _\textbf{x}\phi _i^{(0)}+\hat{\Phi }_i\nabla _\textbf{x}\phi _e^{(0)}+\tilde{\phi }_{i}, \end{aligned}$$

27b $$\begin{aligned}&\phi _e^{(1)}=\Phi _e\nabla _\textbf{x}\phi _e^{(0)}+\hat{\Phi }_e\nabla _\textbf{x}\phi _i^{(0)}+\tilde{\phi }_{e}, \end{aligned}$$

where we have the vectors $\Phi _i$, $\Phi _e$, $\hat{\Phi }_i$ and $\hat{\Phi }_e$ and the scalars $\tilde{\phi }_{i}$ and $\tilde{\phi }_{e}$. The auxiliary vector and scalar fields $\Phi _i$, $\Phi _e$, $\hat{\Phi }_i$, $\hat{\Phi }_e$, $\tilde{\phi }_{i}$ and $\tilde{\phi }_{e}$ satisfy the cell problems

28a $$\begin{aligned}&\nabla _\textbf{y} \cdot (\nabla _\textbf{y}\Phi _i{\textsf{G}}_{i}^{\textrm{T}})+\nabla _\textbf{y}\cdot {\textsf{G}}_{i}^{\textrm{T}}=0\qquad \text{ in }\quad \Omega _i, \end{aligned}$$

28b $$\begin{aligned}&\nabla _\textbf{y} \cdot (\nabla _\textbf{y}\hat{\Phi }_e{\textsf{G}}_{e}^{\textrm{T}})=0\qquad \text{ in }\quad \Omega _e, \end{aligned}$$

28c $$\begin{aligned}&\Phi _{i}=\hat{\Phi }_e \qquad \text{ on }\quad \Gamma , \end{aligned}$$

28d $$\begin{aligned}&(\nabla _\textbf{y}\Phi _i{\textsf{G}}_{i}^{\textrm{T}}-\nabla _\textbf{y}\hat{\Phi }_e{\textsf{G}}_{e}^{\textrm{T}})\cdot \textbf{n}=-{\textsf{G}}_{i}^{\textrm{T}}\cdot \textbf{n}\qquad \text{ on }\quad \Gamma , \end{aligned}$$

and

29a $$\begin{aligned}&\nabla _\textbf{y} \cdot (\nabla _\textbf{y}\hat{\Phi }_i{\textsf{G}}_{i}^{\textrm{T}})=0\qquad \text{ in }\quad \Omega _i, \end{aligned}$$

29b $$\begin{aligned}&\nabla _\textbf{y} \cdot (\nabla _\textbf{y}{\Phi }_e{\textsf{G}}_{e}^{\textrm{T}})+\nabla _\textbf{y}\cdot {\textsf{G}}_{e}^{\textrm{T}}=0\qquad \text{ in }\quad \Omega _e, \end{aligned}$$

29c $$\begin{aligned}&\hat{\Phi }_{i}={\Phi }_e\qquad \text{ on }\quad \Gamma , \end{aligned}$$

29d $$\begin{aligned} (\nabla _\textbf{y}\hat{\Phi }_i{\textsf{G}}_{i}^{\textrm{T}}-\nabla _\textbf{y}{\Phi }_e{\textsf{G}}_{e}^{\textrm{T}})\cdot \textbf{n}={\textsf{G}}_{e}^{\textrm{T}}\cdot \textbf{n}\qquad \text{ on }\quad \Gamma , \end{aligned}$$

and

30a $$\begin{aligned}&\nabla _\textbf{y} \cdot ({\textsf{G}}_{i}\nabla _\textbf{y}{\tilde{\phi }}_i)=0\qquad \text{ in }\quad \Omega _i, \end{aligned}$$

30b $$\begin{aligned}&\nabla _\textbf{y} \cdot ({\textsf{G}}_{e}\nabla _\textbf{y}{\tilde{\phi }}_e)=0\qquad \text{ in }\quad \Omega _e, \end{aligned}$$

30c $$\begin{aligned}&{\tilde{\phi }}_{i}-{\tilde{\phi }}_e=V^{(1)}\qquad \text{ on }\quad \Gamma , \end{aligned}$$

30d $$\begin{aligned} ({\textsf{G}}_{i}\nabla _\textbf{y}{\tilde{\phi }}_i)\cdot \textbf{n}\nonumber \quad =({\textsf{G}}_{e}\nabla _\textbf{y}{\tilde{\phi }}_e)\cdot \textbf{n}\qquad \text{ on }\quad \Gamma . \end{aligned}$$

The above cell problems are also supplemented by periodic conditions on the boundary $\partial \Omega \setminus \Gamma$. For uniqueness of solution, we require a further condition on the auxiliary fields $\Phi _i$, $\Phi _e$, $\hat{\Phi }_i$, $\hat{\Phi }_e$, $\tilde{\phi }_{i}$ and $\tilde{\phi }_{e}$. The condition we propose is the zero average on their individual domains, that is,

31 $$\begin{aligned} \begin{aligned}&\langle \Phi _i\rangle _i=0, \quad \langle \Phi _e\rangle _e=0,\quad \langle \hat{\Phi }_i\rangle _i=0, \quad \langle \hat{\Phi }_e\rangle _e=0, \\&\quad \langle {\tilde{\phi }}_i\rangle _i=0, \quad \langle {\tilde{\phi }}_e\rangle _e=0. \end{aligned} \end{aligned}$$

The ansatz has given expressions for $\phi _i^{(1)}$ and $\phi _e^{(1)}$ (17b) and (17c) and these can be used to write Ohm’s law, Eqs. (25c) and (25d) as

32 $$\begin{aligned} \textbf{j}_i^{(0)}&=-{\textsf{G}}_{i}\nabla _\textbf{x}\phi _i^{(0)}-{\textsf{G}}_{i}\nabla _\textbf{y}\phi _i^{(1)}\nonumber \\&=-({\textsf{G}}_{i}+{\textsf{G}}_{i}(\nabla _\textbf{y}\Phi _i)^{\textrm{T}})\nabla _\textbf{x}\phi _i^{(0)}-{\textsf{G}}_{i}(\nabla _\textbf{y}\hat{\Phi }_i)^{\textrm{T}}\nabla _\textbf{x}\phi _e^{(0)}\nonumber \\&\quad -{\textsf{G}}_{i}\nabla _\textbf{y}\tilde{\phi }_i\nonumber \\&=-({\textsf{G}}_{i}+{\textsf{G}}_{i}{\textsf{R}}_i)\nabla _\textbf{x}\phi _i^{(0)}-({\textsf{G}}_{i}{\textsf{Q}}_i)\nabla _\textbf{x}\phi _e^{(0)}-{\textsf{G}}_{i} \textbf{s}_i \end{aligned}$$

and

33 $$\begin{aligned} \textbf{j}_e^{(0)}&=-{\textsf{G}}_{e}\nabla _\textbf{x}\phi _e^{(0)}-{\textsf{G}}_{e}\nabla _\textbf{y}\phi _e^{(1)}\nonumber \\&=-({\textsf{G}}_{e}+{\textsf{G}}_{e}(\nabla _\textbf{y}\Phi _e)^{\textrm{T}})\nabla _\textbf{x}\phi _e^{(0)}-{\textsf{G}}_{e}(\nabla _\textbf{y}\hat{\Phi }_e)^{\textrm{T}}\nabla _\textbf{x}\phi _i^{(0)}\nonumber \\&\quad -{\textsf{G}}_{e}\nabla _\textbf{y}\tilde{\phi }_e\nonumber \\&=-({\textsf{G}}_{e}+{\textsf{G}}_{e}{\textsf{R}}_e)\nabla _\textbf{x}\phi _e^{(0)}-({\textsf{G}}_{e}{\textsf{Q}}_e)\nabla _\textbf{x}\phi _i^{(0)}-{\textsf{G}}_{e} \textbf{s}_e \end{aligned}$$

with the notation

34 $$\begin{aligned} \begin{aligned} {\textsf{R}}_i&=(\nabla _\textbf{y}\Phi _i)^{\textrm{T}}, \quad {\textsf{R}}_e=(\nabla _\textbf{y}\Phi _e)^{\textrm{T}}, \quad {\textsf{Q}}_i=(\nabla _\textbf{y}\hat{\Phi }_i)^{\textrm{T}}, \\&\quad {\textsf{Q}}_e=(\nabla _\textbf{y}\hat{\Phi }_e)^{\textrm{T}},\quad \textbf{s}_i =\nabla _\textbf{y}\tilde{\phi }_i, \quad \textbf{s}_e=\nabla _\textbf{y}\tilde{\phi }_e. \end{aligned} \end{aligned}$$

The macroscale model requires a balance equation for the current densities. In order to obtain this, we need to equate further powers of epsilon in the multiple scales expansion. We need the coefficient of $\epsilon ^2$ in (15a) and (15b) as well as the $\epsilon ^2$ terms of (15c) and (15d). We have

35a $$\begin{aligned}&\nabla _\textbf{x}\cdot ({\textsf{G}}_{i}\nabla _\textbf{x}\phi _i^{(0)})+\nabla _\textbf{x}\cdot ({\textsf{G}}_{i}\nabla _\textbf{y}\phi _i^{(1)})+\nabla _\textbf{y}\cdot ({\textsf{G}}_{i}\nabla _\textbf{x}\phi _i^{(1)})&\nonumber \\&\quad +\nabla _\textbf{y}\cdot ({\textsf{G}}_{i}\nabla _\textbf{y}\phi _i^{(2)})={\hat{\beta }} (\phi _i^{(0)}-\phi _e^{(0)}), \end{aligned}$$

35b $$\begin{aligned}&\nabla _\textbf{x}\cdot ({\textsf{G}}_{e}\nabla _\textbf{x}\phi _e^{(0)})+\nabla _\textbf{x}\cdot ({\textsf{G}}_{e}\nabla _\textbf{y}\phi _e^{(1)})+\nabla _\textbf{y}\cdot ({\textsf{G}}_{e}\nabla _\textbf{x}\phi _e^{(1)})&\nonumber \\&\quad +\nabla _\textbf{y}\cdot ({\textsf{G}}_{e}\nabla _\textbf{y}\phi _e^{(2)})=-{\hat{\beta }} (\phi _i^{(0)}-\phi _e^{(0)}), \end{aligned}$$

and the coefficients of $\epsilon ^2$ in the expansion of Ohm’s law give

36a $$\begin{aligned}&\textbf{j}_i^{(1)}=-{\textsf{G}}_{i}\nabla _\textbf{x}\phi _i^{(1)} -{\textsf{G}}_{i}\nabla _\textbf{y}\phi _i^{(2)}, \end{aligned}$$

36b $$\begin{aligned}&\textbf{j}_e^{(1)}=-{\textsf{G}}_{e}\nabla _\textbf{x}\phi _e^{(1)} -{\textsf{G}}_{e}\nabla _\textbf{y}\phi _e^{(2)}. \end{aligned}$$

Now by using the $\epsilon ^2$ expansions (36a) and (36b), along with (25c) and (25d) in the $\epsilon ^2$ balance Eqs. (35a) and (35b) we obtain

37a $$\begin{aligned}&\nabla _\textbf{x}\cdot \textbf{j}_i^{(0)}+\nabla _\textbf{y}\cdot \textbf{j}_i^{(1)} ={\hat{\beta }} (\phi _i^{(0)}-\phi _e^{(0)}), \end{aligned}$$

37b $$\begin{aligned}&\nabla _\textbf{x}\cdot \textbf{j}_e^{(0)}+\nabla _\textbf{y}\cdot \textbf{j}_e^{(1)} =-{\hat{\beta }} (\phi _i^{(0)}-\phi _e^{(0)}). \end{aligned}$$

We also consider the $\epsilon ^2$ expansions of interface condition (15m)

38a $$\begin{aligned} ({\textsf{G}}_{i}\nabla _\textbf{x}\phi _i^{(1)} +{\textsf{G}}_{i}\nabla _\textbf{y}\phi _i^{(2)})\cdot \textbf{n}=({\textsf{G}}_{e}\nabla _\textbf{x}\phi _e^{(1)} +{\textsf{G}}_{e}\nabla _\textbf{y}\phi _e^{(2)})\cdot \textbf{n},&\end{aligned}$$

which when using (36a) and (36b) can be written as

39a $$\begin{aligned} \textbf{j}_i^{(1)}\cdot \textbf{n}=\textbf{j}_e^{(1)}\cdot \textbf{n},&\end{aligned}$$

We can now take the sum of (37a) and (37b) and apply the integral average to obtain

40 $$\begin{aligned}&\int _{\Omega _i}\nabla _\textbf{x}\cdot \textbf{j}_i^{(0)}\text{ d }\textbf{y}+\int _{\Omega _i}\nabla _\textbf{y}\cdot \textbf{j}_i^{(1)}\text{ d }\textbf{y}-\int _{\Omega _i}{\hat{\beta }}(\phi _i^{(0)}-\phi _e^{(0)})\text{ d }\textbf{y}\nonumber \\&\quad +\int _{\Omega _e}\nabla _\textbf{x}\cdot \textbf{j}_e^{(0)}\text{ d } \textbf{y} \nonumber \\&\quad +\int _{\Omega _e}\nabla _\textbf{y}\cdot \textbf{j}_e^{(1)}\text{ d }\textbf{y}+\int _{\Omega _e}{\hat{\beta }}(\phi _i^{(0)}-\phi _e^{(0)})\text{ d }\textbf{y}=0 \end{aligned}$$

By applying the assumption of macroscopic uniformity to the first and third integrals and by also applying the Gauss divergence theorem to the second and fourth integrals, we obtain

41 $$\begin{aligned}&\nabla _\textbf{x}\cdot \langle \textbf{j}_i^{(0)}\rangle _i +\nabla _\textbf{x}\cdot \langle \textbf{j}_e^{(0)}\rangle _e+\int _{\partial \Omega _i\setminus \Gamma }\textbf{j}_i^{(1)} \textbf{n}_{\partial \Omega _i}\text{ dS }\nonumber \\&\quad +\int _{\Gamma }\textbf{j}_i^{(1)}\textbf{n}\text{ dS }+\int _{\partial \Omega _e\setminus \Gamma }\textbf{j}_e^{(1)} \textbf{n}_{\partial \Omega _e}\text{ dS }\nonumber \\&\quad -\int _{\Gamma } \textbf{j}_e^{(1)}\textbf{n}\text{ dS }-\int _{\Omega _i}{\hat{\beta }}(\phi _i^{(0)}-\phi _e^{(0)})\text{ d }\textbf{y}\nonumber \\&\quad +\int _{\Omega _e}{\hat{\beta }}(\phi _i^{(0)}-\phi _e^{(0)})\text{ d }\textbf{y}=0. \end{aligned}$$

Since we have the assumption of periodicity the terms on the external boundaries will disappear and by using (39a), we see that the terms on the interface $\Gamma$ will also disappear giving

42 $$\begin{aligned}&\nabla _\textbf{x}\cdot \langle \textbf{j}_i^{(0)}\rangle _i +\nabla _\textbf{x}\cdot \langle \textbf{j}_e^{(0)}\rangle _e-\int _{\Omega _i}{\hat{\beta }}(\phi _i^{(0)}-\phi _e^{(0)})\text{ d }\textbf{y}\nonumber \\&\quad +\int _{\Omega _e}{\hat{\beta }}(\phi _i^{(0)}-\phi _e^{(0)})\text{ d }\textbf{y}=0. \end{aligned}$$

Since we have that the difference in the potentials is $V^{(0)}$ from (17n) and we know that the potentials depend only on the macroscale from (23a) and (23b), we can rewrite (42) as

43 $$\begin{aligned} \nabla _\textbf{x}\cdot \langle \textbf{j}_i^{(0)}\rangle _i +\nabla _\textbf{x}\cdot \langle \textbf{j}_e^{(0)}\rangle _e-{\hat{\beta }} V^{(0)}(|\Omega _e|-|\Omega _i|)=0. \end{aligned}$$

where the averaged leading-order current densities are given as

44a $$\begin{aligned} \langle \textbf{j}_i^{(0)}\rangle _i&=-\langle {\textsf{G}}_{i}+{\textsf{G}}_{i}{\textsf{R}}_i\rangle _i\nabla _\textbf{x}\phi _i^{(0)}-\langle {\textsf{G}}_{i}{\textsf{Q}}_i\rangle _i\nabla _\textbf{x}\phi _e^{(0)}-\langle {\textsf{G}}_i\textbf{s}_i\rangle _i \end{aligned}$$

44b $$\begin{aligned} \langle \textbf{j}_e^{(0)}\rangle _e&=-\langle {\textsf{G}}_{e}+{\textsf{G}}_{e}{\textsf{R}}_e\rangle _e\nabla _\textbf{x}\phi _e^{(0)}-\langle {\textsf{G}}_{e}{\textsf{Q}}_e\rangle _e\nabla _\textbf{x}\phi _i^{(0)}-\langle {\textsf{G}}_e\textbf{s}_e\rangle _e \end{aligned}$$

### Poroelastic problem

We now require a problem for $\textbf{u}_i^{(1)}$ and $\textbf{u}_e^{(1)}$, so by taking (24a), (24b) with (25g) and (25h) along with the interface conditions (17o) and (25p) we can write

45a $$\begin{aligned}&\nabla _y \cdot ({\mathbb {C}}_{i}\zeta _y(\textbf{u}_{i}^{(1)}))=-\nabla _y\cdot ({\mathbb {C}}_{i}\zeta _x(\textbf{u}^{(0)}))+\nabla _y\cdot ({p}^{(0)}\varvec{\alpha }_{i}) ~~ \text{ in } ~\Omega _{i}, \end{aligned}$$

45b $$\begin{aligned}&\nabla _y \cdot ({\mathbb {C}}_{e}\zeta _y(\textbf{u}_{e}^{(1)}))=-\nabla _y\cdot ({\mathbb {C}}_{e}\zeta _x(\textbf{u}^{(0)}))+\nabla _y\cdot ({p}^{(0)}\varvec{\alpha }_{e})~~ \text{ in } ~~ \Omega _{e}, \end{aligned}$$

45c $$\begin{aligned}&({\mathbb {C}}_{i}\zeta _y(\textbf{u}_{i}^{(1)})-{\mathbb {C}}_{e}\zeta _y(\textbf{u}_{e}^{(1)}))\textbf{n}\nonumber \\&\quad =(({\mathbb {C}}_{e}-{\mathbb {C}}_{i})\zeta _x (\textbf{u}^{(0)}) -(\varvec{\alpha }_{e}-\varvec{\alpha }_{i}){p}^{(0)})\textbf{n}~~ \text{ on } ~~\Gamma , \end{aligned}$$

45d $$\begin{aligned}&\textbf{u}_{e}^{(1)}=\textbf{u}_{i}^{(1)}&~~ \text{ on } ~~ \Gamma . \end{aligned}$$

The problem (45a–45d) has a unique solution up to a $\textbf{y}$ constant function. By exploiting linearity, we propose the ansatz

46a $$\begin{aligned}&\textbf{u}_{i}^{(1)}={\mathcal {B}}_{i}\zeta _x(\textbf{u}^{(0)})+\textbf{b}_{i}{p}^{(0)}+c_1(\textbf{x}), \end{aligned}$$

46b $$\begin{aligned}&\textbf{u}_{e}^{(1)}={\mathcal {B}}_{e}\zeta _x(\textbf{u}^{(0)})+\textbf{b}_{e}{p}^{(0)}+c_2(\textbf{x}), \end{aligned}$$

where we have that $c_1(\textbf{x})$ and $c_2(\textbf{x})$ are $\textbf{y}$ constant functions and we have the third-order tensors ${\mathcal {B}}_{i}$ and ${\mathcal {B}}_{e}$ which satisfy the cell problems given by

47a $$\begin{aligned}&\nabla _y \cdot ({\mathbb {C}}_{i}\zeta _y({\mathcal {B}}_{i}))=-\nabla _y\cdot {\mathbb {C}}_{i}\qquad \text{ in } \quad \Omega _{i}, \end{aligned}$$

47b $$\begin{aligned}&\nabla _y \cdot ({\mathbb {C}}_{e}\zeta _y({\mathcal {B}}_{e}))=-\nabla _y\cdot {\mathbb {C}}_{e}\qquad \text{ in } \quad \Omega _{e}, \end{aligned}$$

47c $$\begin{aligned}&({\mathbb {C}}_{i}\zeta _y({\mathcal {B}}_{i})-{\mathbb {C}}_{e}\zeta _y({\mathcal {B}}_{e}))\textbf{n}=({\mathbb {C}}_{e}-{\mathbb {C}}_{i})\textbf{n}\qquad \text{ on } \quad \Gamma , \end{aligned}$$

47d $$\begin{aligned}&{\mathcal {B}}_{i}={\mathcal {B}}_{e}\qquad \text{ on } \quad \Gamma , \end{aligned}$$

as well as the vectors $\textbf{b}_{i}$ and $\textbf{b}_{e}$ which satisfy the problem

48a $$\begin{aligned}&\nabla _y \cdot ({\mathbb {C}}_{i}\zeta _y(\textbf{b}_{i}))=\nabla _y\cdot \varvec{\alpha }_{i}\qquad \text{ in } \quad \Omega _{i}, \end{aligned}$$

48b $$\begin{aligned}&\nabla _y \cdot ({\mathbb {C}}_{e}\zeta _y(\textbf{b}_{e}))=\nabla _y\cdot \varvec{\alpha }_{e}\qquad \text{ in } \quad \Omega _{e}, \end{aligned}$$

48c $$\begin{aligned}&({\mathbb {C}}_{i}\zeta _y(\textbf{b}_{i})-{\mathbb {C}}_{e}\zeta _y(\textbf{b}_{e}))\textbf{n}=-(\varvec{\alpha }_{i}-\varvec{\alpha }_{e})\textbf{n}\qquad \text{ on } \quad \Gamma , \end{aligned}$$

48d $$\begin{aligned}&\textbf{b}_{i}=\textbf{b}_{e}\qquad \text{ on } \quad \Gamma . \end{aligned}$$

The problems (47a–47d) and (48a–48d) are further supplemented with periodic conditions on $\partial \Omega \setminus \Gamma$. To ensure the uniqueness of the solution, we require one further condition on the auxiliary variables ${\mathcal {B}}_{i}$, ${\mathcal {B}}_{e}$, $\textbf{b}_{i}$ and $\textbf{b}_{e}$, and we impose zero average on the individual subsets of the domain. That is,

49 $$\begin{aligned} \langle {\mathcal {B}}_{i}\rangle _{i}=0, \quad \langle {\mathcal {B}}_{e}\rangle _{e}=0, \quad \langle \textbf{b}_{i}\rangle _i=0, \quad \langle \textbf{b}_{e}\rangle _{e}=0. \end{aligned}$$

Now that we have expressions for the leading-order elastic displacements, we can write the leading-order effective stress tensors in the myocyte and matrix, respectively, as

50 $$\begin{aligned} {\textsf{T}}_{i}^{(0)}&={\mathbb {C}}_{i}\zeta _y({\mathcal {B}}_{i}\zeta _x(\textbf{u}^{(0)})+\textbf{b}_{i}{p}^{(0)})\nonumber \\&\quad +{\mathbb {C}}_{i}\zeta _x(\textbf{u}^{(0)})-\varvec{\alpha }_{i}p^{(0)} \nonumber =({\mathbb {C}}_{i}{\mathbb {L}}_{i}+{\mathbb {C}}_{i})\zeta _x(\textbf{u}^{(0)})+({\mathbb {C}}_{i}{\varvec{\tau }}_{i}-\varvec{\alpha }_{i}){p}^{(0)}, \end{aligned}$$

where we have the auxiliary tensors

51 $$\begin{aligned} {\mathbb {L}}_{i}=\zeta _y({\mathcal {B}}_{i}) \qquad \text{ and } \qquad {\varvec{\tau }}_{i}=\zeta _y(\textbf{b}_{i}), \end{aligned}$$

and

52 $$\begin{aligned} {\textsf{T}}_{e}^{(0)}&={\mathbb {C}}_{e}\zeta _y({\mathcal {B}}_{e}\zeta _x(\textbf{u}^{(0)})+\textbf{b}_{e}{p}^{(0)})+{\mathbb {C}}_{e}\zeta _x(\textbf{u}^{(0)})-\varvec{\alpha }_{e}p^{(0)} \nonumber \\&=({\mathbb {C}}_{e}{\mathbb {L}}_{e}+{\mathbb {C}}_{e})\zeta _x(\textbf{u}^{(0)})+({\mathbb {C}}_{e}{\varvec{\tau }}_{e}-\varvec{\alpha }_{e}){p}^{(0)}, \end{aligned}$$

where we have used the notation

53 $$\begin{aligned} {\mathbb {L}}_{i}=\zeta _y({\mathcal {B}}_{i}) \qquad \text{ and } \qquad {\varvec{\tau }}_{i}=\zeta _y(\textbf{b}_{i}). \end{aligned}$$

The tensor and vector terms appearing in (51), and analogously in (53), are the microscale gradients of the auxiliary tensors and vectors that are solving the microscale poroelastic cell problems (47a)–(47d) and (48a)–(48d). This means that they are capturing the microscale variations in different geometries and physical properties of the phases and encoding them in the macroscale model. We can describe the fourth rank tensors ${\mathbb {L}}_i$ and ${\mathbb {L}}_e$ as correction terms to the average of the effective elasticity tensors, thus accounting for differences in elastic properties at different points in the microstructure. The second rank tensors $\varvec{\tau }_i$ and $\varvec{\tau }_e$ are accounting for the variations in the compressibility of the microstructure at different points.

We require a balance equation for the effective stresses that takes into consideration both the myocytes and the extracellular matrix. We apply the integral average to (25e) and (25f) to obtain

54 $$\begin{aligned}&\int _{\Omega _i}\nabla _\textbf{x}\cdot {\textsf{T}}_i^{(0)}\text{ d }\textbf{y}+\int _{\Omega _i}\nabla _\textbf{y}\cdot {\textsf{T}}_i^{(1)}\text{ d } \textbf{y} +\int _{\Omega _i}({\textsf{G}}_i\nabla _\textbf{x}\phi _i^{(0)}\times \textbf{B}^{(0)})\text{ d } \textbf{y}\nonumber \\&\quad +\int _{\Omega _i}({\textsf{G}}_i\nabla _\textbf{y}\phi _i^{(1)}\times \textbf{B}^{(0)})\text{ d } \textbf{y}+\int _{\Omega _e}\nabla _\textbf{x}\cdot {\textsf{T}}_e^{(0)}\text{ d } \textbf{y}\nonumber \\&\quad +\int _{\Omega _e}\nabla _\textbf{y}\cdot {\textsf{T}}_e^{(1)}\text{ d } \textbf{y}\nonumber \\&\quad +\int _{\Omega _e}({\textsf{G}}_e\nabla _\textbf{x}\phi _e^{(0)}\times \textbf{B}^{(0)})\text{ d } \textbf{y}\nonumber \quad+\int _{\Omega _e}({\textsf{G}}_e\nabla _\textbf{y}\phi _e^{(1)}\times \textbf{B}^{(0)})\text{ d } \textbf{y}=0. \end{aligned}$$

We apply the divergence theorem to the second and seventh integral and use the expressions we have for the first-order electric potentials (27a) and (27b) that we have for $\phi _i^{(1)}$ and $\phi _e^{(1)}$ to write

55 $$\begin{aligned}&\nabla _\textbf{x}\cdot \langle {\textsf{T}}_i^{(0)}\rangle _i+\nabla _\textbf{x}\cdot \langle {\textsf{T}}_e^{(0)}\rangle _e+\int _{\partial \Omega _i\setminus \Gamma } {\textsf{T}}_i^{(1)}\cdot \textbf{n}_{\partial \Omega _i}\text{ dS }\nonumber \\&\quad -\int _{\Gamma } {\textsf{T}}_i^{(1)}\cdot \textbf{n}\text{ dS }\nonumber \\&\quad +\int _{\partial \Omega _e\setminus \Gamma } {\textsf{T}}_e^{(1)}\cdot \textbf{n}_{\partial \Omega _e}\text{ dS }+\int _{\Gamma } {\textsf{T}}_e^{(1)}\cdot \textbf{n}\text{ dS }\nonumber \\&\quad +\int _{\Omega _i}({\textsf{G}}_i\nabla _\textbf{x}\phi _i^{(0)}\times \textbf{B}^{(0)})\text{ d } \textbf{y}\nonumber \\&\quad +\int _{\Omega _e}({\textsf{G}}_e\nabla _\textbf{x}\phi _e^{(0)}\times \textbf{B}^{(0)})\text{ d } \textbf{y}+\int _{\Omega _i}({\textsf{G}}_i\nabla _\textbf{y}(\Phi _i\nabla _\textbf{x}\phi _i^{(0)}\nonumber \\&\quad +\hat{\Phi }_i\nabla _\textbf{x}\phi _e^{(0)}+\tilde{\phi }_i)\times \textbf{B}^{(0)})\text{ d } \textbf{y}\nonumber \\&\quad +\int _{\Omega _e}({\textsf{G}}_e\nabla _\textbf{y}(\Phi _e\nabla _\textbf{x}\phi _e^{(0)}+\hat{\Phi }_e\nabla _\textbf{x}\phi _i^{(0)}+\tilde{\phi }_e)\times \textbf{B}^{(0)})\text{ d }\textbf{y}=0. \end{aligned}$$

Due to periodicity, the terms of the external boundaries cancel and the terms on $\Gamma$ cancel due to the interface condition (25o). This means that we have

56 $$\begin{aligned}&\nabla _\textbf{x}\cdot \langle {\textsf{T}}_i^{(0)}\rangle _i+\nabla _\textbf{x}\cdot \langle {\textsf{T}}_e^{(0)}\rangle _e+\int _{\Omega _i}({\textsf{G}}_i\nabla _\textbf{x}\phi _i^{(0)}\times \textbf{B}^{(0)})\text{ d } \textbf{y}\nonumber \\&+\int _{\Omega _e}({\textsf{G}}_e\nabla _\textbf{x}\phi _e^{(0)}\times \textbf{B}^{(0)})\text{ d } \textbf{y}+\int _{\Omega _i}({\textsf{G}}_i\mathsf \nabla _\textbf{y}\Phi _i\nabla _\textbf{x}\phi _i^{(0)}\times \textbf{B}^{(0)})\text{ d } \textbf{y}\nonumber \\&+\int _{\Omega _i}({\textsf{G}}_i\nabla _\textbf{y}\hat{\Phi }_i\nabla _\textbf{x}\phi _e^{(0)}\times \textbf{B}^{(0)})\text{ d } \textbf{y}+\int _{\Omega _i}({\textsf{G}}_i\nabla _\textbf{y}{\tilde{\phi }}_i\times \textbf{B}^{(0)})\text{ d } \textbf{y}\nonumber \\&+\int _{\Omega _e}({\textsf{G}}_e\nabla _\textbf{y}\Phi _e\nabla _\textbf{x}\phi _e^{(0)}\times \textbf{B}^{(0)})\text{ d } \textbf{y}+\int _{\Omega _e}({\textsf{G}}_e\nabla _\textbf{y}\hat{\Phi }_e\nabla _\textbf{x}\phi _i^{(0)}\nonumber \\&\quad \times \textbf{B}^{(0)})\text{ d } \textbf{y}\nonumber \\&+\int _{\Omega _e}({\textsf{G}}_e\nabla _\textbf{y}{\tilde{\phi }}_e\times \textbf{B}^{(0)})\text{ d }\textbf{y}=0. \end{aligned}$$

We can rewrite using the integral average notation, and using (34) and rearranging, we have

57 $$\begin{aligned}&\nabla _\textbf{x}\cdot \langle {\textsf{T}}_i^{(0)}\rangle _i+\nabla _\textbf{x}\cdot \langle {\textsf{T}}_e^{(0)}\rangle _e=-\langle {\textsf{G}}_i {\textsf{R}}_i+{\textsf{G}}_i\rangle _i\nabla _\textbf{x}\phi _i^{(0)}\times \langle \textbf{B}^{(0)}\rangle _i\nonumber \\&-\langle {\textsf{G}}_e{\textsf{R}}_e+{\textsf{G}}_e\rangle _e\nabla _\textbf{x}\phi _e^{(0)}\times \langle \textbf{B}^{(0)}\rangle _e-\langle {\textsf{G}}_i{\textsf{Q}}_i\rangle _i\nabla _\textbf{x}\phi _e^{(0)}\times \langle \textbf{B}^{(0)}\rangle _i\nonumber \\&-\langle {\textsf{G}}_e\mathsf {Q_e}\rangle _e\nabla _\textbf{x}\phi _i^{(0)}{\times }\langle \textbf{B}^{(0)}\rangle _e-\langle {\textsf{G}}_i\textbf{s}_i{\times }\textbf{B}^{(0)}\rangle _i-\langle {\textsf{G}}_e\textbf{s}_e{\times }\textbf{B}^{(0)}\rangle _e \end{aligned}$$

### The Darcy flow problem

We can use the balance Eqs. (17k) and (17l) with the interface conditions (17q) and (25r) to write the following problem for the first-order pressures in each phase $p_{i}^{(1)}$ and $p_{e}^{(1)}$

58a $$\begin{aligned}&\nabla _y \cdot \textbf{w}_{i}^{(0)}=0\quad \text{ in } \quad \Omega _{i}, \end{aligned}$$

58b $$\begin{aligned}&\nabla _y \cdot \textbf{w}_{e}^{(0)}=0\quad \text{ in } \quad \Omega _{e}, \end{aligned}$$

58c $$\begin{aligned}&p_{e}^{(1)}=p_{e}^{(1)}&\quad \text{ on }\quad \Gamma , \end{aligned}$$

58d $$\begin{aligned}&\textbf{w}_{i}^{(0)}\cdot \textbf{n}=\textbf{w}_{e}^{(0)}\cdot \textbf{n}\quad \text{ on }\quad \Gamma . \end{aligned}$$

Using the expressions (25i) and (25j) that we have for $\textbf{w}_{i}^{(0)}$ and $\textbf{w}_{e}^{(0)},$ we rewrite the problem (58a–58d) in terms of the pressures

59a $$\begin{aligned}&\nabla _y \cdot ({{\textsf{K}}}_{i}\nabla _y p_{i}^{(1)})=-\nabla _y\cdot ({{\textsf{K}}}_{i}\nabla _x{p}^{(0)})\quad \text{ in } ~~\Omega _{i}, \end{aligned}$$

59b $$\begin{aligned}&\nabla _y \cdot ({{\textsf{K}}}_{e}\nabla _yp_{e}^{(1)})=-\nabla _y\cdot ({{\textsf{K}}}_{e}\nabla _x{p}^{(0)})\quad \text{ in }~~\Omega _{e}, \end{aligned}$$

59c $$\begin{aligned}&p_{i}^{(1)}=p_{e}^{(1)}&\quad \text{ on }~~\Gamma , \end{aligned}$$

59d $$\begin{aligned}&({{\textsf{K}}}_{i}\nabla _y p_{i}^{(1)}-{{\textsf{K}}}_{e}\nabla _y p_{e}^{(1)})\cdot \textbf{n}=({{\textsf{K}}}_{e}-{{\textsf{K}}}_{i})\nabla _x{p}^{(0)}\cdot \textbf{n}\quad \text{ on }~~\Gamma . \end{aligned}$$

This problem then admits a unique solution up to a $\textbf{y}$ constant function (see Cioranescu and Donato 1999; Bakhvalov et al. 1989). By exploiting the linearity, we are able to propose the following ansatz

60a $$\begin{aligned}&p_{i}^{(1)}=\textbf{P}_{i}\cdot \nabla _x{p}^{(0)}+c_3(\textbf{x}), \end{aligned}$$

60b $$\begin{aligned}&p_{e}^{(1)}=\textbf{P}_{e}\cdot \nabla _x{p}^{(0)}+c_4(\textbf{x}), \end{aligned}$$

where we have that $c_3(\textbf{x})$ and $c_4(\textbf{x})$ are $\textbf{y}$ constant functions and the vectors $\textbf{P}_{i}$ and $\textbf{P}_{e}$ satisfy the cell problem given by

61a $$\begin{aligned}&\nabla _y\cdot (\nabla _y\textbf{P}_{i}{{\textsf{K}}}_{i}^{\textrm{T}})=-\nabla _y\cdot {{\textsf{K}}}_{i}^{\textrm{T}}\qquad \text{ in } \quad \Omega _{i}, \end{aligned}$$

61b $$\begin{aligned}&\nabla _y\cdot (\nabla _y\textbf{P}_{e}{{\textsf{K}}}_{e}^{\textrm{T}})=-\nabla _y\cdot {{\textsf{K}}}_{e}^{\textrm{T}}\qquad \text{ in } \quad \Omega _{e}, \end{aligned}$$

61c $$\begin{aligned}&\textbf{P}_{i}=\textbf{P}_{e}\qquad \text{ on }\quad \Gamma , \end{aligned}$$

61d $$\begin{aligned}&(\nabla _y\textbf{P}_{i}{{\textsf{K}}}_{i}^{\textrm{T}}-\nabla _y\textbf{P}_{e}{{\textsf{K}}}_{e}^{\textrm{T}})\textbf{n}=({{\textsf{K}}}_{e}-{{\textsf{K}}}_{i})^{\textrm{T}}\textbf{n}\qquad \text{ on }\quad \Gamma . \end{aligned}$$

The cell problem is supplemented by periodic conditions on the boundary $\partial \Omega \setminus \Gamma ,$ and for the sake of uniqueness of solution, we place a further condition on $\textbf{P}_{i}$ and $\textbf{P}_{e}$. We chose zero average over the subsection of the domain, that is,

62 $$\begin{aligned} \langle \textbf{P}_{i}\rangle _i=0, \quad \text{ and }\quad \langle \textbf{P}_{e}\rangle _{e}=0. \end{aligned}$$

We know wish to find the expressions for the leading-order Darcy’s law in the myocytes and extracellular matrix, respectively. We can use the expressions (60a) and (60b) for the first-order pressures $p_{i}^{(1)}$ and $p_{e}^{(1)}$ in (25i) and (25j), and by applying the integral average, we obtain

63 $$\begin{aligned} \langle \textbf{w}_{i}^{(0)}\rangle _{i}&=-\langle {{\textsf{K}}}_{i}(\nabla _y\textbf{P}_{i})^{\textrm{T}}\rangle _{i}\nabla _x{p}^{(0)}-\langle {{\textsf{K}}}_{i}\rangle _{i}\nabla _x{p}^{(0)}\nonumber \\&=-\langle {{\textsf{K}}}_{i}{{\textsf{R}}}_{i}+{{\textsf{K}}}_{i}\rangle _{i}\nabla _x {p}^{(0)}, \end{aligned}$$

in the myocyte where we have used the notation

64 $$\begin{aligned} {{\textsf{R}}}_{i}=(\nabla _y\textbf{P}_{i})^{\textrm{T}}, \end{aligned}$$

and we obtain

65 $$\begin{aligned} \langle \textbf{w}_{e}^{(0)}\rangle _{e}&=-\langle {{\textsf{K}}}_{e}(\nabla _y\textbf{P}_{e})^{\textrm{T}}\rangle _{e}\nabla _x{p}^{(0)}-\langle {{\textsf{K}}}_{e}\rangle _{e}\nabla _x{p}^{(0)}\nonumber \\&=-\langle {{\textsf{K}}}_{e}{{\textsf{R}}}_{e}+{{\textsf{K}}}_{e}\rangle _{e}\nabla _x {p}^{(0)}, \end{aligned}$$

in the extracellular matrix where we have used the notation

66 $$\begin{aligned} {{\textsf{R}}}_{e}=(\nabla _y\textbf{P}_{e})^{\textrm{T}}. \end{aligned}$$

We now wish to use these to quantities to determine an effective Darcy’s law. We have

67 $$\begin{aligned} \textbf{w}_{\textrm{eff}}:&=\langle \textbf{w}_{i}^{(0)}\rangle _i+\langle \textbf{w}_{e}^{(0)}\rangle _e\nonumber \\ &=-\big (\langle {{\textsf{K}}}_{i}{{\textsf{R}}}_{i}+{{\textsf{K}}}_{i}\rangle _i+\langle {{\textsf{K}}}_{e}{{\textsf{R}}}_{e}+{{\textsf{K}}}_{e}\rangle _e\big )\nabla _x {p}^{(0)}. \end{aligned}$$

Here we can define an effective hydraulic conductivity tensor as

68 $$\begin{aligned} {\textsf{W}}=\langle {{\textsf{K}}}_{i}{{\textsf{R}}}_{i}+{{\textsf{K}}}_{i}\rangle _i+\langle {{\textsf{K}}}_{e}{{\textsf{R}}}_{e}+{{\textsf{K}}}_{e}\rangle _e \end{aligned}$$

and this means that the Darcy’s law can be rewritten as

69 $$\begin{aligned} \textbf{w}_{\textrm{eff}}=-{{\textsf{W}}}\nabla _x {p}^{(0)}. \end{aligned}$$

### Conservation of mass

The final equation we require is the macroscale conservation of mass equation. To obtain this, we integrate the expressions (25k) and (25l), which are the conservation of mass equation in each subdomain, in $\Omega _{i}$ and $\Omega _{e}$, respectively. That is,

70 $$\begin{aligned}&\int _{\Omega _{i}}\frac{{\dot{p}}^{(0)}}{{M}_{i}}\text{ dy }+\int _{\Omega _{e}}\frac{{\dot{p}}^{(0)}}{{M}_{e}}\text{ dy }=-\int _{\Omega _{i}} \varvec{\alpha }_{i}:\zeta _x (\dot{\textbf{u}}^{(0)})\text{ dy }\nonumber \\&\quad -\nabla _x \cdot \int _{\Omega _{i}} \textbf{w}_{i}^{(0)}{\text{ dy }}\nonumber \\&\quad -\int _{\Omega _{e}} \varvec{\alpha }_{e}:\zeta _x (\dot{\textbf{u}}^{(0)})\text{ dy }-\nabla _x \cdot \int _{\Omega _{e}} \textbf{w}_{e}^{(0)} \textbf{dy}\nonumber \\&\quad -\int _{\Omega _{i}} \varvec{\alpha }_{i}:\zeta _y (\dot{\textbf{u}}_{i}^{(1)})\text{ dy }\nonumber \\&\quad -\int _{\Omega _{e}} \varvec{\alpha }_{e}:\zeta _y( \dot{\textbf{u}}_{e}^{(1)})\text{ dy }-\int _{\Omega _{i}} \nabla _y\cdot \textbf{w}_{i}^{(1)}\text{ dy }-\int _{\Omega _{e}} \nabla _y\cdot \textbf{w}_{e}^{(1)}\text{ dy }. \end{aligned}$$

We then apply the divergence theorem and use the interface condition (25q) to cancel the final two terms. This allows us to rewrite the remaining terms as

71 $$\begin{aligned}&\bigg (\frac{\langle {M}_{i}\rangle _i+\langle {M}_{e}\rangle _e}{\langle {M}_{i}\rangle _{i}\langle {M}_{e}\rangle _{e}}\bigg ){\dot{p}}^{(0)}\nonumber \\&\quad =-(\langle \varvec{\alpha }_{i}\rangle _i +\langle \varvec{\alpha }_{e}\rangle _e):\zeta _x(\dot{\textbf{u}}^{(0)})-\nabla _x\cdot ( \langle \textbf{w}_{i}^{(0)}\rangle _i+\langle \textbf{w}_{e}^{(0)}\rangle _e)\nonumber \\&\quad \quad -\langle \varvec{\alpha }_{i}:\zeta _y\left( \dot{\textbf{u}}_{i}^{(1)}\right) \rangle _i-\langle \varvec{\alpha }_{e}:\zeta _y (\dot{\textbf{u}}_{e}^{(1)})\rangle _e. \end{aligned}$$

Since we have the expressions for the leading-order solid displacements $\textbf{u}_{i}^{(1)}$ and $\textbf{u}_{e}^{(1)}$ from (46a) and (46b), we can differentiate these with respect to time to obtain $\dot{\textbf{u}}_{i}^{(1)}$ and $\dot{\textbf{u}}_{e}^{(1)}$ and then use these new expressions in (71) to obtain

72 $$\begin{aligned}&\bigg (\frac{\langle {M}_{i}\rangle _i+\langle {M}_{e}\rangle _e}{\langle {M}_{i}\rangle _{i}\langle {M}_{e}\rangle _{e}}\bigg ){\dot{p}}^{(0)}=-\bigg (\left( \langle \varvec{\alpha }_{i}\rangle _i+\langle \varvec{\alpha }_{e}\rangle _e\right) :\zeta _x(\dot{\textbf{u}}^{(0)})\nonumber \\&\quad +\nabla _x\cdot \textbf{w}_{\textrm{eff}}+\left( \langle {\mathbb {L}}_{i}^{\textrm{T}}:\varvec{\alpha }_{i}\rangle _i+\langle {\mathbb {L}}_{e}^{\textrm{T}}:\varvec{\alpha }_{e}\rangle _e\right) :\zeta _x (\dot{\textbf{u}}^{(0)})\nonumber \\&\quad +(\langle \varvec{\alpha }_{i}:{\varvec{\tau }}_{i}\rangle _i +\langle \varvec{\alpha }_{e}:{\varvec{\tau }}_{e}\rangle _e) {\dot{p}}^{(0)}\bigg ). \end{aligned}$$

We can now rearrange this equation to obtain an expression for ${\dot{p}}^{(0)}$

73 $$\begin{aligned} {\dot{p}}^{(0)}&=-\bar{{\mathcal {M}}}\bigg (\nabla _x\cdot \textbf{w}_{\textrm{eff}}+(\langle \varvec{\alpha }_{i}+{\mathbb {L}}_{i}^{\textrm{T}}:\varvec{\alpha }_{i}\rangle _i\nonumber \\&\quad +\langle \varvec{\alpha }_{e} +{\mathbb {L}}_{e}^{\textrm{T}}: \varvec{\alpha }_{e}\rangle _e):\zeta _x( \dot{\textbf{u}}^{(0)})\bigg ) \end{aligned}$$

where we have use the notation

74 $$\begin{aligned} \bar{{\mathcal {M}}}:=\frac{\langle {M}_{i}\rangle _{i}\langle {M}_{e}\rangle _{e}}{\langle {M}_{i}\rangle _{i}+\langle {M}_{e}\rangle _{e}+\langle {M}_{i}\rangle _{i}\langle {M}_{e}\rangle _{e}(\langle \varvec{\alpha }_{i}:{\varvec{\tau }}_{i}\rangle _{i} +\langle \varvec{\alpha }_{e}:{\varvec{\tau }}_{e}\rangle _{e})}, \end{aligned}$$

which is reminiscent of the Biot’s modulus for the system. We also define a tensor quantity

75 $$\begin{aligned} \bar{\varvec{\alpha }}:=\langle \varvec{\alpha }_{i}+{\mathbb {L}}_{i}^{\textrm{T}}:\varvec{\alpha }_{i}\rangle _i+\langle \varvec{\alpha }_{e} +{\mathbb {L}}_{e}^{\textrm{T}}: \varvec{\alpha }_{e}\rangle _e, \end{aligned}$$

which reminds of an effective Biot’s tensor of coefficients.

We have now derived all the equations required to be able to state our macroscale model.

## Macroscale model

The macroscale equations describe the effective behaviour of the heart in terms of the leading-order elastic displacement $\textbf{u}^{(0)}$, the leading-order electric potentials $\phi _i^{(0)}$ and $\phi _e^{(0)}$, the relative fluid–solid velocity $\textbf{w}_{\textrm{eff}}$ and the pressure $p^{(0)}$. The model is given by

76a $$\begin{aligned}&\nabla _\textbf{x}\cdot \langle \textbf{j}_i^{(0)}\rangle _i +\nabla _\textbf{x}\cdot \langle \textbf{j}_e^{(0)}\rangle _e={\hat{\beta }} V^{(0)}(|\Omega _e|-|\Omega _i|), \end{aligned}$$

76b $$\begin{aligned}&\nabla _\textbf{x}\cdot \langle {\textsf{T}}_i^{(0)}\rangle _i+\nabla _\textbf{x}\cdot \langle {\textsf{T}}_e^{(0)}\rangle _e=-\langle {\textsf{G}}_i {\textsf{R}}_i+{\textsf{G}}_i\rangle _i\nabla _\textbf{x}\phi _i^{(0)}\times \langle \textbf{B}^{(0)}\rangle _i\nonumber \\&-\langle {\textsf{G}}_e{\textsf{R}}_e+{\textsf{G}}_e\rangle _e\nabla _\textbf{x}\phi _e^{(0)}\times \langle \textbf{B}^{(0)}\rangle _e-\langle {\textsf{G}}_i{\textsf{Q}}_i\rangle _i\nabla _\textbf{x}\phi _e^{(0)}\times \langle \textbf{B}^{(0)}\rangle _i\nonumber \\&-\langle {\textsf{G}}_e\mathsf {Q_e}\rangle _e\nabla _\textbf{x}\phi _i^{(0)}{\times }\langle \textbf{B}^{(0)}\rangle _e-\langle {\textsf{G}}_i\textbf{s}_i{\times } \textbf{B}^{(0)}\rangle _i-\langle {\textsf{G}}_e\textbf{s}_e{\times } \textbf{B}^{(0)}\rangle _e \end{aligned}$$

76c $$\begin{aligned}&\phi _i^{(0)}-\phi _e^{(0)}=V^{(0)}, \end{aligned}$$

76d $$\begin{aligned} {\dot{p}}^{(0)}&=-\bar{{\mathcal {M}}}\big (\nabla _x\cdot \textbf{w}_{\textrm{eff}} +(\langle \varvec{\alpha }_{i}+{\mathbb {L}}_{i}^{\textrm{T}}:\varvec{\alpha }_{i}\rangle _i+\langle \varvec{\alpha }_{e}\nonumber \\&\quad +{\mathbb {L}}_{e}^{\textrm{T}}: \varvec{\alpha }_{e}\rangle _e):\zeta _x( \dot{\textbf{u}}^{(0)})\big ) \end{aligned}$$

76e $$\begin{aligned}&\textbf{w}_{\textrm{eff}}=-{\textsf{W}}\nabla _xp^{(0)} \end{aligned}$$

where we have the averaged leading-order current densities

77a $$\begin{aligned} \langle \textbf{j}_i^{(0)}\rangle _i&=-\langle {\textsf{G}}_{i}+{\textsf{G}}_{i}{\textsf{R}}_i\rangle _i\nabla _\textbf{x}\phi _i^{(0)}-\langle {\textsf{G}}_{i}{\textsf{Q}}_i\rangle _i\nabla _\textbf{x}\phi _e^{(0)}-\langle {\textsf{G}}_i\textbf{s}_i\rangle _i \end{aligned}$$

77b $$\begin{aligned} \langle \textbf{j}_e^{(0)}\rangle _e&=-\langle {\textsf{G}}_{e}+{\textsf{G}}_{e}{\textsf{R}}_e\rangle _e\nabla _\textbf{x}\phi _e^{(0)}-\langle {\textsf{G}}_{e}{\textsf{Q}}_e\rangle _e\nabla _\textbf{x}\phi _i^{(0)}-\langle {\textsf{G}}_e\textbf{s}_e\rangle _e \end{aligned}$$

and the averaged leading-order solid stresses

78a $$\begin{aligned}&\langle {\textsf{T}}_i^{(0)}\rangle _i=\langle {\mathbb {C}}_i+{\mathbb {C}}_i{\mathbb {L}}_i\rangle _i\zeta _\textbf{x}(\textbf{u}^{(0)})+\langle {\mathbb {C}}_i\varvec{\tau }_i-\varvec{\alpha }_i\rangle _ip^{(0)} \end{aligned}$$

78b $$\begin{aligned}&\langle {\textsf{T}}_e^{(0)}\rangle _i=\langle {\mathbb {C}}_e+{\mathbb {C}}_e{\mathbb {L}}_e\rangle _e\zeta _\textbf{x}(\textbf{u}^{(0)})+\langle {\mathbb {C}}_e\varvec{\tau }_e-\varvec{\alpha }_e\rangle _ep^{(0)} \end{aligned}$$

The novel PDE model has the balance equation for the leading-order current densities (76a). The leading-order current densities (77a) and (77b) are the sum of the electric fields one from each compartment which are premultiplied by second rank tensors that are to be obtained by solving the cell problems (28a)–(28d) and (29a)–(29d). The current densities also contain a vector term that is the solution to the cell problem (30a)–(30d) which is driven by the difference in the electric potentials. The coefficients arising from solving the cell problem account for the differences in the electric potentials in each phase and encode these in the model.

The macroscale model also possesses a balance equation for the solid stresses (76b), where the stresses are given by (78a) and (78b). These stresses comprise tensors ${\mathbb {C}}_i+{\mathbb {C}}_i{\mathbb {L}}_i$ and ${\mathbb {C}}_e+{\mathbb {C}}_e{\mathbb {L}}_e$ which are found by solving (47a)–(47d). The stresses also contain terms relating to the fluid pressure where the coefficients of these terms are to be found by solving (48a)–(48d). The problems to be solved are similar to those found for elastic composite in Penta and Gerisch (2015, 2017) and poroelastic composites in Miller and Penta (2020, 2023, 2022). The balance equation (76b) also has terms that relate to the electric potentials and Lorentz forces on the deformations of the material. These terms are to be found by solving the electric cell problems (28a)–(28d) and (29a)–(29d) and (30a)–(30d). This equation tells us how the changes in the elastic deformations influence the mechanotransduction of the myocardium.

Equation (76c) provides a constraint such that the $V^{(0)}$ is a given and therefore allows that only one of $\phi _i^{(0}$ or $\phi _e^{(0)}$ is to be calculated in order to obtain both.

We have (76d) which is the conservation of mass equation. This equation comprises the divergence of the relative fluid–solid velocity and the Biot’s tensor of coefficients applied to the leading-order strains. The Biot’s tensor that we obtain here (75) comprises the Biot’s tensors from the myocyte and the extracellular matrix as well as the two additional contributions arising due to the fact that we are accounting for changing compressibility at different points on the microstructure.

And finally we have Darcy’s law (76e) with the modified hydraulic conductivity tensor ${\textsf{W}}$ (68). This tensor consists of the hydraulic conductivities ${\textsf{K}}_i$ and ${\textsf{K}}_e$ from the myocyte and extracellular matrix as well as two additional terms. The extra terms are ${\textsf{K}}_i{\textsf{R}}_i$ and ${\textsf{K}}_e{\textsf{R}}_e$ and these are accounting for the differences in the hydraulic conductivities at different points in the microstructure. The hydraulic conductivity tensor ${\textsf{W}}$ can be found by solving cell problem (61a)–(61d).

Our new macroscale model (76a)–(76e) describes the effective electrostatic and mechanical behaviour of a double poroelastic material subjected to a magnetic Lorentz force representing the myocardium. The model incorporates fine scale behaviours through the coefficients which are solved via the specific cell problems referenced in the preceding paragraphs. The key novelties of the model are that (1) the macroscale coefficients encode the differences in microstructure over two finer scales of resolution, and (2) it encodes the difference in poroelastic and electrical properties/moduli at different points in the microstructure. Our macroscale stress balance equation captures how the elastic displacement of the myocyte and extracellular matrix are driven by the applied magnetic fields. We also note that the cell problems are all fully decoupled from each other. This means that we are solving the electrical problems (28a)–(28d) and (29a)–(29d) and (30a)–(30d) fully separately from the poroelastic problems (47a)–(47d) and (48a)–(48d), which are again fully separated from the Darcy flow problems (61a)–(61d). Therefore, we also note that the fluid that fills the poroelastic matrix is there only as a contribution via a porosity since the simulations are drained. In order to understand what details this model is capturing, in the next section we wish to numerically investigate the behaviour of the electrical and poroelastic coefficients of the model.

## Numerical simulations

**Fig. 2:**
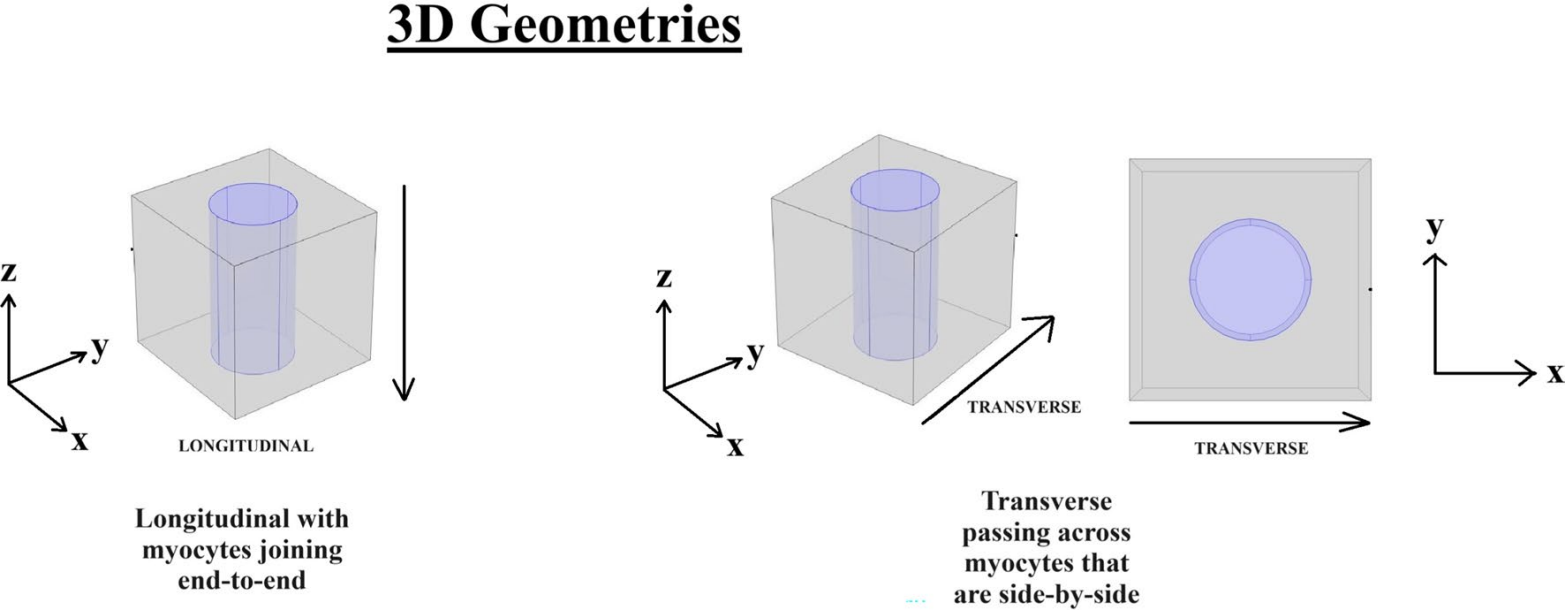
COMSOL Multiphysics 3D geometries showing the direction of the myocyte elongation as well as the direction of the electrical conductances

Here we investigate the coefficients of our novel model. We consider both the electrostatic and poroelastic cell problems. We first investigate the effective electrical conductance tensor that arises from the balance equation and secondly consider the double poroelastic simulations. As the 3D geometry of our unit cell is assumed to be a cube with cylindrical myocyte extending in the z-axis direction, we are able to cut the plane and carry out 2D simulations (see Fig. 2) which will be less computationally expensive while still retaining the desired accuracy, see Parnell and Abrahams (2006, 2008); Miller and Penta (2022) for a reduction of poroelastic-type cell problems from 3D to 2D and validation of the 2D simulations.

### Electrical simulations

In the macroscale model, we have the balance equation for the leading-order current densities (76a). This can be rewritten as follows when using (77a) and (77b)

79 $$\begin{aligned}&\nabla _\textbf{x}\cdot \bigg (\big (\langle {\textsf{G}}_{i}+{\textsf{G}}_{i}{\textsf{R}}_i\rangle _i +\langle {\textsf{G}}_e{\textsf{Q}}_e\rangle _e\big )\nabla _\textbf{x}\phi _i^{(0)}\nonumber \\&\quad +\big (\langle {\textsf{G}}_{e} +{\textsf{G}}_{e}{\textsf{R}}_e\rangle _e+\langle {\textsf{G}}_i{\textsf{Q}}_i\rangle _i\big )\nabla _\textbf{x}\phi _e^{(0)}\nonumber \\&\quad +\big (\langle {\textsf{G}}_i\textbf{s}_i\rangle _i+\langle {\textsf{G}}_e\textbf{s}_e\rangle _e\big ) \bigg )=-{\hat{\beta }} V^{(0)}(|\Omega _e|-|\Omega _i|). \end{aligned}$$

Since we want to investigate the effective electrostatic properties of the material, it is useful to define an effective conductance tensor. To do this, we want to write as a diffusion-type equation. We therefore can substitute the macroscale equation (76c) written in the form $\phi _i^{(0)}=\phi _e^{(0)}+V^{(0)}$ into (79) and rearrange to obtain

80 $$\begin{aligned}&\nabla _\textbf{x}\cdot \Big (\big (\langle {\textsf{G}}_{i}+{\textsf{G}}_{i}{\textsf{R}}_i\rangle _i+\langle {\textsf{G}}_e{\textsf{Q}}_e\rangle _e +\langle {\textsf{G}}_{e}+{\textsf{G}}_{e}{\textsf{R}}_e\rangle _e\nonumber \\&\qquad +\langle {\textsf{G}}_i{\textsf{Q}}_i\rangle _i\big )\nabla _\textbf{x}\phi _e^{(0)}\Big )\nonumber \\&\quad =\nabla _\textbf{x} \cdot \Big (-\big (\langle {\textsf{G}}_{i}+{\textsf{G}}_{i}{\textsf{R}}_i\rangle _i+\langle {\textsf{G}}_e{\textsf{Q}}_e\rangle _e\big )\nabla _\textbf{x}V^{(0)}-\langle {\textsf{G}}_i\textbf{s}_i\rangle _i\nonumber \\&\qquad -\langle {\textsf{G}}_e\textbf{s}_e\rangle _e\Big )\nonumber -{\hat{\beta }} V^{(0)}(|\Omega _e|-|\Omega _i|) \end{aligned}$$

We can define the following

81 $$\begin{aligned} {\textsf{D}}:&=\langle {\textsf{G}}_{i}+{\textsf{G}}_{i}{\textsf{R}}_i\rangle _i+\langle {\textsf{G}}_e{\textsf{Q}}_e\rangle _e+\langle {\textsf{G}}_{e}+{\textsf{G}}_{e}{\textsf{R}}_e\rangle _e+\langle {\textsf{G}}_i{\textsf{Q}}_i\rangle _i, \end{aligned}$$

82 $$\begin{aligned} \textbf{f}:&=-\big (\langle {\textsf{G}}_{i}+{\textsf{G}}_{i}{\textsf{R}}_i\rangle _i+\langle {\textsf{G}}_e{\textsf{Q}}_e\rangle _e\big )\nabla _\textbf{x}V^{(0)}-\langle {\textsf{G}}_i\textbf{s}_i\rangle _i-\langle {\textsf{G}}_e\textbf{s}_e\rangle _e, \end{aligned}$$

83 $$\begin{aligned} \tilde{\beta }:&={\hat{\beta }}(|\Omega _e|-|\Omega _i|), \end{aligned}$$

and therefore, (80) can be written as

84 $$\begin{aligned} \nabla _\textbf{x}\cdot \bigg ({\textsf{D}}\nabla _\textbf{x}\phi _e^{(0)}\bigg ) =\nabla _\textbf{x} \cdot \textbf{f}-\tilde{\beta }V^{(0)}. \end{aligned}$$

We can then solve the cell problems (28a)–(28d) and (29a)–(29d) to determine the second rank tensor ${\textsf{D}}$ which we call the effective conductance tensor.

We are considering the effective conductance tensor of the myocardium and we wish to consider how it is influenced by structural changes related to heart diseases such as myocardial infarction or growth and remodelling. It can physiologically be observed that post-myocardial infarction, the volume fraction of myocytes in the infarct zone decreases due to the death and damage of myocytes. This dramatically reduces the functionality of the heart as the damaged myocytes are replaced by a thick collagen scar. The heart requires to function normally and therefore looks for ways to compensate for the scar tissue. In this case, and in the case of most tissue remodelling, it is a well-known homeostasis mechanism for healthy regions of the myocardium to have an increase in myocyte volume to attempt to compensate for heart diseases (Olivetti et al. 1987). We therefore choose to focus on the general effect that the change in myocyte volume has on the electrical conductivity of the myocardium. We should note that we are assuming that the increases in myocyte volume fraction that we are studying correspond to the extent of remodelling and are not time dependent (Olivetti et al. 1994; Anversa et al. 1985).

**Fig. 3:**
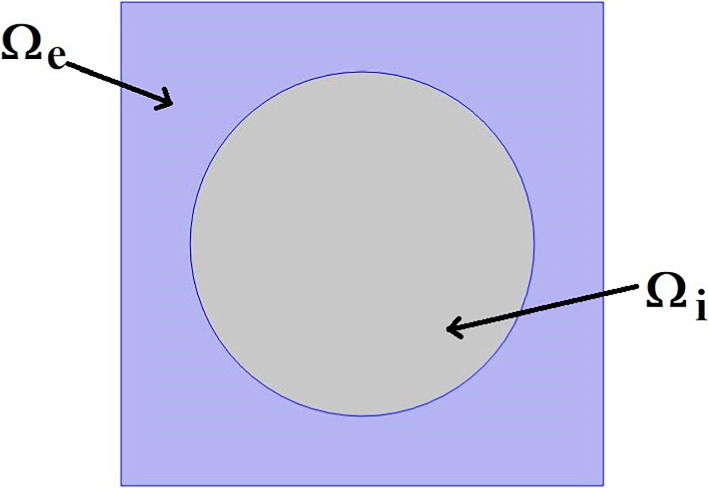
COMSOL Multiphysics geometry for the electrostatic cell problems

The cell problems are solved on the following 2D composite geometry (Fig. 3).

We use the following conductivity tensors obtained from Roth (1991); Sachse et al. (2009) for the transversal and longitudinal conductivities in the myocyte and extracellular matrix

85 $$\begin{aligned} {\textsf{G}}_i=\begin{pmatrix} 0.047 & 0 \\ 0 & 0.469 \end{pmatrix}, \quad \text{ and } \quad {\textsf{G}}_e=\begin{pmatrix} 0.214 & 0 \\ 0 & 0.375 \end{pmatrix}. \end{aligned}$$

We consider the two components of the second rank tensor ${\textsf{D}}$ in the balance Eq. (84).

**Fig. 4:**
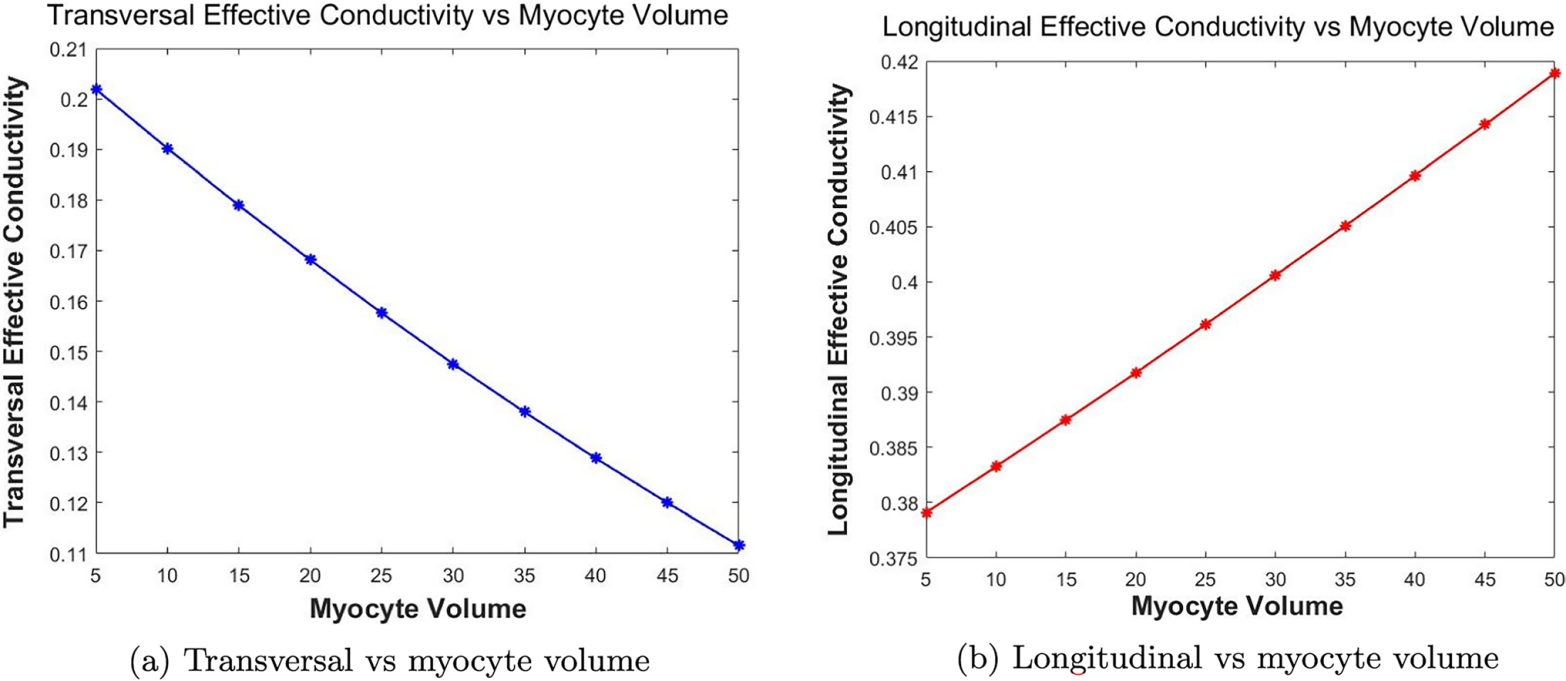
Plots of the components of the second rank effective conductivity tensor ${\textsf{D}}$

Figure 4a shows the transverse component of our effective conductivity tensor ${\textsf{D}}$ decreases with the increasing myocyte volume. We can explain this due to the fact that the myocyte has a much lower conductivity in the transversal direction than the extracellular matrix, see input experimental parameters (85), and therefore, as the volume of the myocyte increases the matrix volume decreases, and so, the value of the myocyte takes over leading to the decrease in the transversal conductance. Figure 4b shows that the longitudinal component of the effective conductivity increases with increasing myocyte volume. This can be explained due to the fact that the myocyte has already a higher longitudinal conductance than the extracellular matrix, see input experimental parameters (85), and as it increases in volume, this larger value plays an increasingly important role in the conductance of ${\textsf{D}}$. The myocytes in adjacent cells join end to end; therefore, the larger the volume fraction of the myocyte, the larger the contact area between adjacent myocytes is likely leading to the increasing longitudinal conductance.

### Poroelastic simulations

**Fig. 5:**
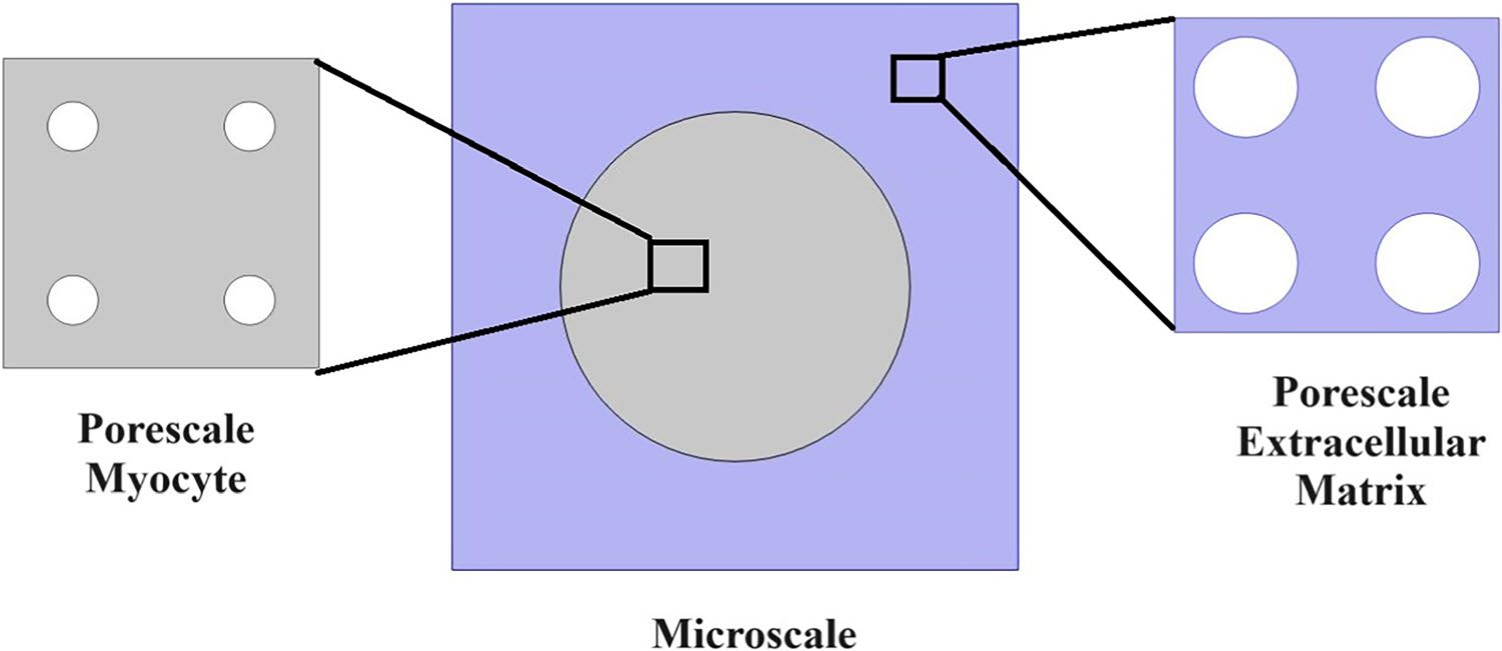
COMSOL Multiphysics geometries for the double poroelastic material

In this section, we wish to consider the poroelastic response of the myocardium. The model tells us that the poroelastic behaviour can be fully described by the material’s effective elasticity tensor, the hydraulic conductivity tensor ${\textsf{W}}$, the tensor $\bar{\varvec{\alpha }}$ which is reminiscent of the classical Biot’s tensor of coefficients and the scalar quantity $\bar{{\mathcal {M}}}$ which can be identified with the Biot’s modulus. The relevant cell problems to find the poroelastic coefficients are (47a)–(47d) and (48a)–(48d) and (61a)–(61d).

As we noted above, we are considering the case where we have an increase in myocyte volume as a homeostasis mechanism in heart disease and remodelling. We therefore wish to investigate the influence that this change in volume has on the overall elastic parameters of the heart.

Within this subsection, we make the assumption that the bundles of myocytes run from the top of the cell to the bottom as a single cylindrical fibre. The myocytes here are approximated as cylinders. This means that we can cut the plane and perform 2D simulations to solve the cell problems (47a)–(47d), (48a)–(48d) and (61a)–(61d).

As we are dealing with a double poroelastic material, our simulations will be a two-step process. Figure 5 shows the composite made of the two porous media. These are the 2D geometries that we perform the simulations on in COMSOL Multiphysics. Before we can solve the composite problems ((47a)–(47d), (48a)–(48d) and (61a)–(61d)), we must find the input parameters for the myocyte and the extracellular matrix by solving the problems for a porous matrix as done in Dehghani et al. (2018). We assume that the matrix and the extracellular matrix have different porosities and elastic parameters and we obtain ${\mathbb {C}}_i$, ${\mathbb {C}}_e$, $\varvec{\alpha }_i$, $\varvec{\alpha }_e$, ${\textsf{K}}_i$, ${\textsf{K}}_e$, $M_i$ and $M_e$. These are the input parameters for solving the cell problems (47a)–(47d), (48a)–(48d) and (61a)–(61d) and also form part of the poroelastic coefficients. The results of the simulations to find the input parameters are found in Appendix.

We will begin by considering the effective elasticity tensor which we can define as

86 $$\begin{aligned} \tilde{{\mathbb {C}}}=\langle {\mathbb {C}}_i+{\mathbb {C}}_i{\mathbb {L}}_i\rangle _i+\langle {\mathbb {C}}_e+{\mathbb {C}}_e{\mathbb {L}}_e\rangle _e \end{aligned}$$

Due to the geometry, we are assuming for the microstructure we are including the effects of anisotropy of the myocardium tissue in our results. This means that we have more than one independent shear and more than one independent Young’s modulus. Our material is not fully orthotropic with three Young’s moduli and three shears since there is a symmetry in *x* and *y*. Therefore, due to the symmetries imposed by our choice of geometry we should note that the shear $C_{44}$ is the same as the shear $C_{55}$, so we consider shears $C_{44}$ and $C_{66}$. We also only have the two Young’s moduli $E_1$ and $E_3$, since $E_1$ is the same as $E_2$. We carry out the simulations for four fixed total underlying porosities (low porosity, small porosity, mid-porosity and high porosity) and for each of these varying the myocyte volume fraction from $10 \, \text{to} \, 60\%$.

We first consider the two independent shears.

**Fig. 6:**
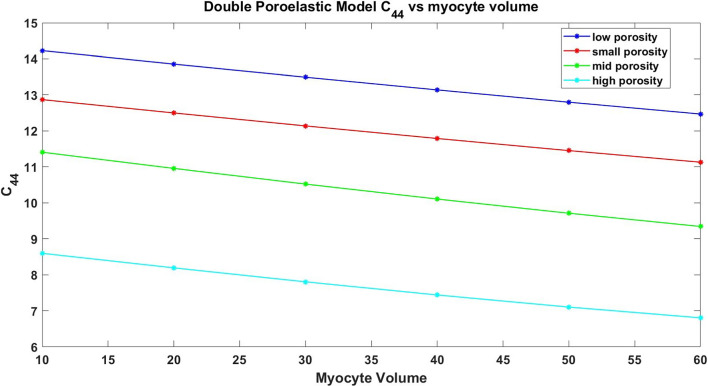
Shear $C_{44}$ vs increasing myocyte volume

Figure 6 shows that the shear $C_{44}$ decreases with increasing myocyte volume fraction. In the case of $C_{44}$, the force is being applied in the axial direction, and this is the direction in which the myocytes elongate. We can deduce that it is likely that since the myocyte is softer than the extracellular matrix then the larger it gets the easier it is for the material to deform. We also believe this is the reason that in the case where we have the lowest porosity (i.e. the matrix and myocyte are at their stiffest) we see the largest value of shear. Indeed as the porosity of the phases increases they will get softer and this coupled with the increase in the volume of the softer myocyte explains the decrease we see in the figure.

**Fig. 7:**
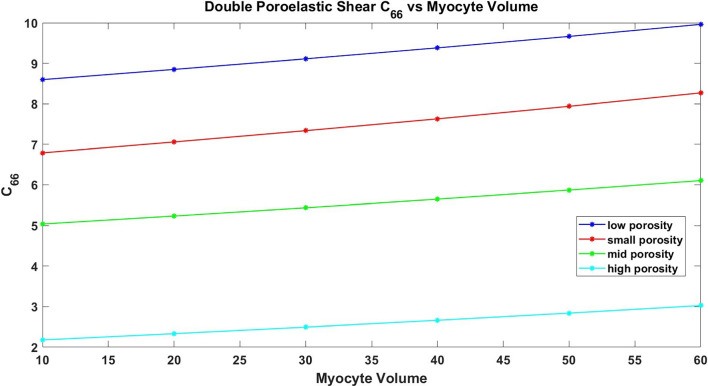
Shear $C_{66}$ vs increasing myocyte volume

Figure 7 shows that shears actually increase with the increasing myocyte volume fraction. For $C_{66},$ the force is being applied in the transverse direction, that is, the force is being applied taking a cross section of the structure where we have the myocyte. The increase in the stiffness can likely be explained by the direction in which we apply the force. As the myocyte increases in volume we have a material that is reinforced by a very large fibre, this leads to the resistance and indeed the increase in stiffness. The higher the porosity, the less the effect this fibre has in reinforcing the material as it itself will be soft.

We also wish to consider the comparison between the two Young’s moduli $E_{1}$ (transverse) and $E_{3}$ (axial). We compute the components of the effective elasticity tensor and use in the formulas for the Young’s moduli. These formulas, which can derived via inverting the elasticity tensor and comparing with the material compliance tensor (Vignjevic et al. 2008), are given by

87 $$\begin{aligned} E_{1}&=\frac{(C_{12}-C_{11})(2C_{13}^{2}-C_{12}C_{33}-C_{11}C_{33})}{(-C^2_{13}+C_{11}C_{33})} \end{aligned}$$

88 $$\begin{aligned} E_{3}&=\frac{(2C^2_{13}-C_{12}C_{33}-C_{11}C_{33})}{(-C_{12}-C_{11})} \end{aligned}$$

**Fig. 8:**
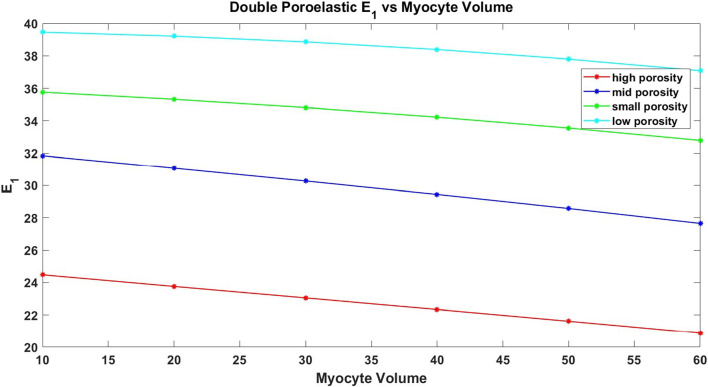
Young’s modulus $E_1$ vs increasing myocyte volume

Figure 8 shows that the transverse Young’s modulus $E_1$ decreases with increasing myocyte volume fraction and this behaviour is consistent across the four porosity levels that we have considered. The Young’s modulus can be thought of as a measure of material stiffness, so in the case of low myocyte volume fraction the extracellular matrix is the dominating parameter in influencing the stiffness of the overall material. A stiffer material leads to less elastic compliance. So we see that as the myocyte, which is softer, gets larger than the compliance of the material is indeed improved; this also makes sense as to why the higher the porosity of the phases is then the more compliant the overall double poroelastic material is.

**Fig. 9:**
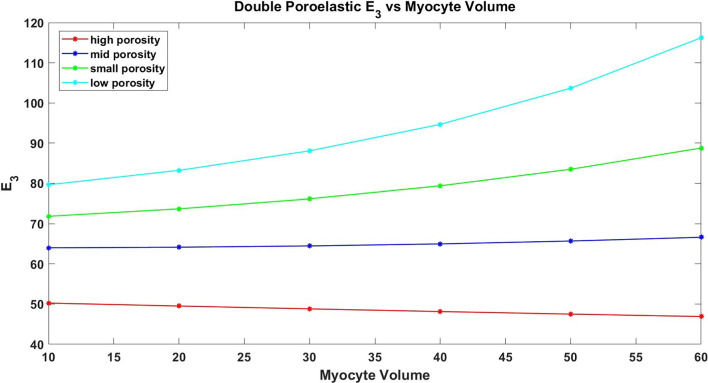
Young’s modulus $E_3$ vs increasing myocyte volume

Figure 9 shows that the values of axial Young’s modulus $E_3$ increase for increasing myocyte volume fraction. This can likely be explained due to the fact that this is the direction that the myocytes elongate in. This means that as the fibres increase in volume they give more resistance when pulling the material. This also agrees with the fact that the largest values are seen for the case where the underlying porosity of both phases is the lowest.

We also wish to consider the Biot’s modulus for the double poroelastic material. We have

89 $$\begin{aligned} \bar{{\mathcal {M}}}:=\frac{\langle {M}_{i}\rangle _{i}\langle {M}_{e}\rangle _{e}}{\langle {M}_{i}\rangle _{i}+\langle {M}_{e}\rangle _{e}+\langle {M}_{i}\rangle _{i}\langle {M}_{e}\rangle _{e}(\langle \varvec{\alpha }_{i}:{\varvec{\tau }}_{i}\rangle _{i} +\langle \varvec{\alpha }_{e}:{\varvec{\tau }}_{e}\rangle _{e})}, \end{aligned}$$

This can be found by using the Biot’s moduli $M_i$ and $M_e$ that we obtain for each porous matrix as well as the results $\varvec{\tau }_i$ and $\varvec{\tau }_e$ obtained from solving (48a)–(48d). The results of the simulations to find $M_i$ and $M_e$ are presented in Appendix.

To find $\varvec{\tau }_i$ and $\varvec{\tau }_{e},$ we need to solve the cell problem (48a)–(48d); to do this, we require the Biot’s tensors of coefficients $\varvec{\alpha }_i$ and $\varvec{\alpha }_e$ which are calculated in the myocyte and extracellular matrix. The results of the simulations to find are presented in Appendix.

Using the values of $\varvec{\alpha }_i$ and $\varvec{\alpha }_{e},$ we can then solve the cell problem (48a)–(48d). The solution to this cell problem can then be used with $M_i$ and $M_e$ to obtain the Biot’s modulus for a double poroelastic material $\bar{{\mathcal {M}}}$ (89) which we have plotted against increasing porosity.

**Fig. 10:**
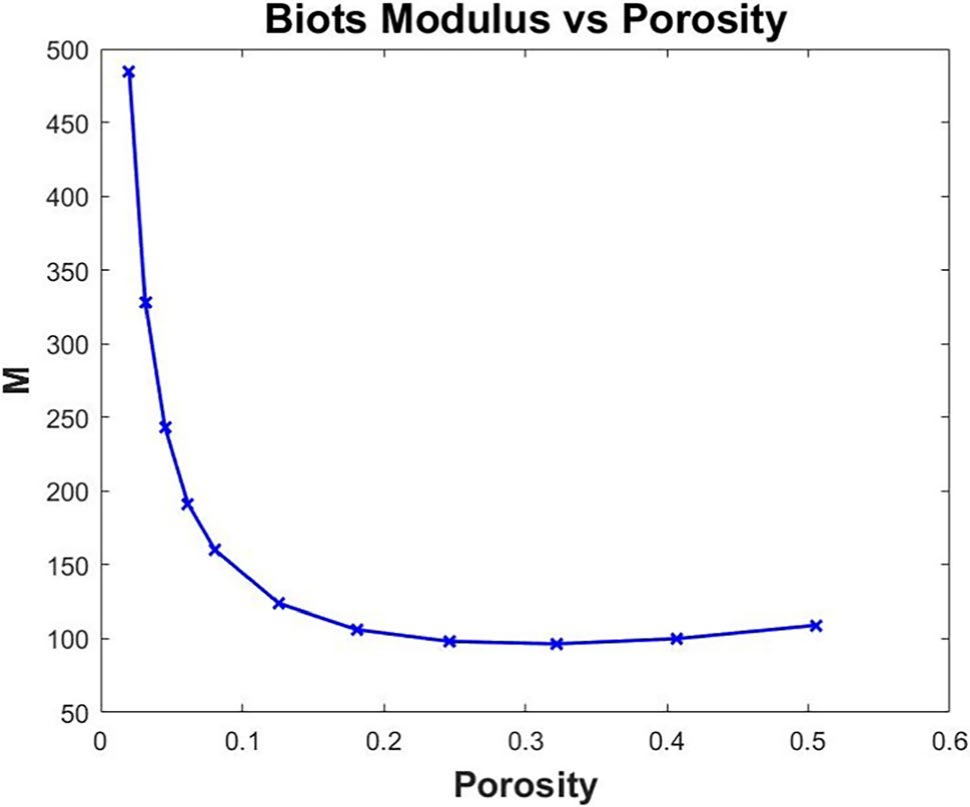
Biot’s modulus vs increasing porosity

Figure 10 shows that the effective Biot’s modulus versus porosity exhibits an unusual behaviour. The results of our numerical simulations show that the Biot’s modulus is initially decreasing for increasing porosity but then begins to increase again. This can likely be explained via the fact that the Biot’s modulus comprises the Biot’s modulus of the individual phases as well as the additional terms that account for local changes in the compressibility.

## Conclusion

This work has led to the derivation of a novel system of PDEs that describes the effective electrical and mechanical behaviour of a poroelastic composite that represents the heart muscle. The structure consists of a poroelastic extracellular matrix with embedded poroelastic myocytes, and we consider the mechanical and electrical interactions between them.

To derive the novel system of macroscale PDEs, we set up a problem that described the electrostatic and poroelastic interactions that occur between the cardiac myocytes and the extracellular matrix. We consider a resolution of the microstructure where we can visibly see the myocytes and matrix distinctly resolved from each other. If we zoom in further on both the myocytes and the extracellular matrix, then we find that each domain can be governed by Biot’s poroelasticity due to their underlying porous nature. The difference in scales allows us to apply the asymptotic homogenization technique to upscale the microstructural problem, accounting for the continuity of current densities, stresses, elastic displacements, fluxes and pressures as well as the difference in the electric potentials across the interface. The novel macroscale PDEs contains balance equations for the current densities and stresses as well as a conservation of mass equation and a modified Darcy’s law. The model coefficients encode properties of the microstructure to be retained in the macroscale model, and these are to be computed by solving the microscale differential problems that arise during the upscaling.

The novel model in this work is an extension to the previously presented electrical and mechanical bidomain model of Miller and Penta (2023) and the model of double poroelastic materials (Miller and Penta 2021a) by combining them to create an electrical and mechanical myocardium model accounting for the fact that the myocytes and matrix have an underlying poroelastic nature. This encodes an extra level of microstructural details to the final macroscale model and additionally allows for a greater understanding of the myocardial behaviour due to a more realistic microstructure being considered. The key novelties of the model are that (1) the macroscale coefficients encode the differences in microstructure over two finer scales of resolution and (2) it encodes the difference in poroelastic and electrical properties/moduli at different points in the microstructure via the solution of the cell problems. Our macroscale stress balance equation captures how the elastic displacement of the myocyte and extracellular matrix are driven by the applied magnetic fields. The microscale cell problems presented in this work for the electrostatic terms (28a)–(28d), (29a)–(29d) and (30a)–(30d) and the double poroelastic terms (47a)–(47d) and (48a)–(48d) and (61a)–(61d) have been solved to provide the first analysis of the effective conductivity tensor and the elastic and poroelastic properties of the myocardium.

By modelling the electrostatic activity of the heart, this can give rise to better understanding of how the electrostatic function is impaired or changed by various cardiac diseases. In the case of cardiac ischaemia, we find there is a change in the cardiac action potentials and the membrane potential increases with a larger uptake of potassium ions. The combination of these features means that our macroscale model can investigate how structural changes caused by myocardial ischaemia affects heart electrophysiology.

The current model is subject to some limitations that can be addressed in future works. Here we have assumed that the myocytes and extracellular matrix are governed by Biot’s linear poroelasticity. However, it would be possible to extend this and use a nonlinear poroelastic formulation for each of the phases, such as the large deformation poroelastic models of Brown et al. (2014); Collis et al. (2017) or the nonlinear poroelastic composite model (Miller and Penta 2021b). This, however, would increase the computational complexity as the length scales between the pore and microscales in these models remain coupled and this means that the cell problems have a huge computational expense to be solved. However, advances are being made to overcome the complexity (Dehghani and Zilian 2021; Dehghani and Zilian 2023). We could also obtain results by using a piecewise linear approach as done in Hu et al. (2003a, b). By doing this, we can approximate the nonlinear behaviour using simple and computationally cheap simulations. We have also governed the domains using passive steady-state equations. It would be a very useful extension to this work to consider active stresses and active strain such as in Pezzuto and Ambrosi (2014); Pezzuto et al. (2014) as this would allow for more realistic computations of the heart actively beating and undergoing deformation. We also note that the current work assumes that the material is homogeneous, and therefore, macroscopic uniformity (see Remark 2) can be applied. This assumption can be relaxed and there are various methods discussed in the literature to account for the case where the microstructure varies with respect to the macroscale point (Penta et al. 2014; Burridge and Keller 1981; Holmes 2012; Penta and Gerisch 2015; Dalwadi et al. 2015).

In the current work, we have that the difference in potentials $V^{(0)}$ is assumed to be a given. As this $V^{(0)}$ or indeed $V^{(1)}$, that drives the cell problem (30a)–(30d) arises due to transport of ions at a finer microstructural level than we are considering in this work, it would be possible to create a finer scale problem to obtain an expression for these values and cell problems from which they can be calculated.

In the future, this work could be developed in a variety of ways. A potential theoretical extension will be to couple with a vascular network, such as in Penta and Merodio (2017), as this will provide a much more realistic microstructure for the myocardium. This addition would then also be expandable to transport of solute between domains such as to investigate drug delivery to the myocardium. It is also necessary to investigate whether the assumption of the fluid being Newtonian is always realistic. In the case of small vessels/pores, it would be appropriate to consider a more complex rheology; however, in vessels with a much larger radius, then blood can be treated as a continuum with an approximately constant viscosity. It would also be possible to consider the effects of growth and remodelling post-infarction or disease spread (Penta et al. 2014; O’Dea et al. 2010; Wang et al. 2017). We will also solve the macroscale system of equations presented in this work to understand the behaviour of the myocardium that our model can capture such as the magnetically driven elastic displacements. This is an area which is currently being explored as an alternative use of MRI imaging (Roth et al. 2014), where the contrast dye does not need to be injected to the patient preimaging. We also would like to validate the current work against experimental data to give an insight into how this computationally feasible myocardium model could have clinical utility as a diagnostic tool.

## Figures and Tables

**Fig. 11:**
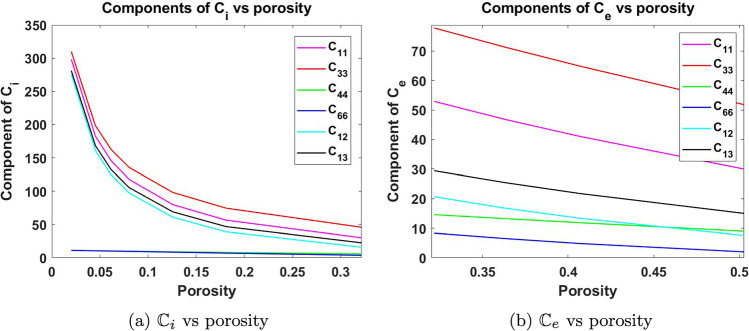
Components of the elasticity tensors vs porosity

**Fig. 12:**
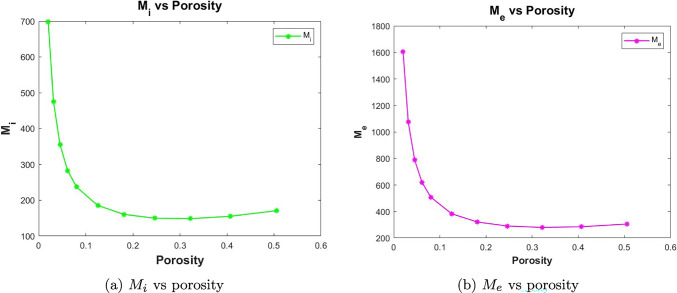
Biot’s moduli vs porosity

**Fig. 13:**
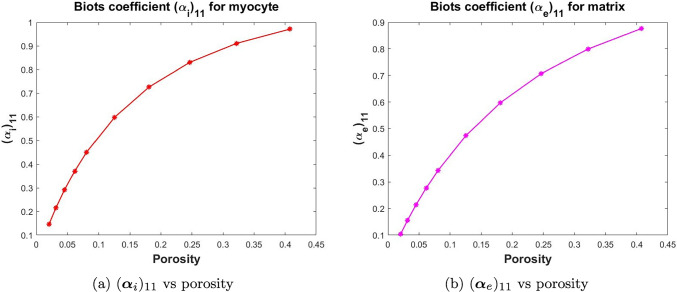
Eleven component of the Biot’s tensor of coefficients vs porosity

## Data Availability

There are no additional data supporting this study other than those already reported in the manuscript and its appendices.

## Appendix A

### A.1 The asymptotic homogenization technique

Here we introduce the asymptotic homogenization technique. This will be used to derive the macroscale model for Eqs. (13a–13r). To apply this technique, we must first assume that the microscale length denoted by *d* is very small in comparison with the average size of the domain *L*. That is,

90 $$\begin{aligned} \epsilon =\frac{d}{L} \ll 1. \end{aligned}$$

Due to this scale separation, we must introduce a local spatial variable that will capture microscale variations of each of the fields in (13a−13r), we have

91 $$\begin{aligned} \textbf{y}=\frac{\textbf{x}}{\epsilon }. \end{aligned}$$

We note that the spatial variables $\textbf{x}$ and $\textbf{y}$ should be considered formally independent where $\textbf{x}$ represents the macroscale and $\textbf{y}$ the microscale. The gradient operator is transformed via application of the chain rule as

92 $$\begin{aligned} \nabla \rightarrow \nabla _x+\frac{1}{\epsilon }\nabla _y. \end{aligned}$$

We further assume that all the fields, as well as the elasticity tensors are functions of both $\textbf{x}$ and $\textbf{y}$. We also assume that the fields can be represented in terms of a series expansion in powers of $\epsilon$, i.e.

93 $$\begin{aligned} \varphi ^{\epsilon }(\textbf{x,y},t)=\sum _{l=0}^\infty \varphi ^{(l)}(\textbf{x,y},t)\epsilon ^l, \end{aligned}$$

where we have used $\varphi$ to denote a general field involved in the present analysis in (13a−13r). For simplicity, we make the assumption that the difference in the electric potentials *V* is a given and has the following multiple scale expansion

94 $$\begin{aligned} V=V^{(0)}(\textbf{x}, t)+\cdots . \end{aligned}$$

We see that this means it depends only on the macroscale at order zero. This assumption has also been made in Richardson and Chapman (2011).

In order to progress further with the model derivation, we must also make the following two assumptions.

#### Remark 1

(Microscale Periodicity) The microstructure of materials is vast, and therefore, in order to create computationally feasible yet realistic models we must simplify the analysis that will be carried out. In this work, we consider the myocardium which has many structural features; we, however, restrict our focus to a single subset of the myocardium which we call the periodic cell which contains a myocyte phase and an extracellular matrix phase. In order to carry out the analysis on this periodic cell, we need to assume that every field $\varphi ^{(l)}$ in our problem (13a)–(13r) is $\textbf{y}$-periodic. This assumption means that it will be the case that the microscale differential problems arising from applying the asymptotic homogenization technique are to be solved on the periodic cell of our material. It is not necessary to make this assumption, and an analysis can be carried out under the assumption of local boundedness of fields. The local boundedness approach allows only the determination of the functional form of the macroscale model. The coefficients of the model derived in this form are related to microscale problems that are to be solved on the whole material microstructure. This is a very computationally expensive approach in comparison with microscale periodicity. Some examples of local boundedness are illustrated in Burridge and Keller (1981); Penta and Gerisch (2017).

#### Remark 2

(Macroscopic Uniformity) In multiscale materials, it is a known fact that for each macroscale point the underlying microstructure may well be different. The variation in microstructure is each macroscale point is a subject of much interest and has been investigated by Penta et al. (2014); Burridge and Keller (1981); Holmes (2012); Penta and Gerisch (2015); Dalwadi et al. (2015). By considering that the microscale will depend on the macroscale, this will add additional terms to the final model. These terms arise via proper application of the Reynolds transport theorem. This, however, leads to much greater computational complexity and cost. For this reason, in this work we will assume that at every macroscale point the microstructure will be the same. This means that the microscale geometry does not depend on the variable $\textbf{x}$. We define this property as macroscopic uniformity and it will be employed in this work. This allows for the simple differentiation under the integral sign

95 $$\begin{aligned} \int _{\Omega }\nabla _x \cdot (\cdot)\text{ dy }=\nabla _x\cdot \int _{\Omega }(\cdot)\text{ dy }, \end{aligned}$$

where $(\cdot)$ is a tensor or a vector quantity.

#### Remark 3

(Periodic Cell) Before beginning the analysis, we make the identification between the domain $\Omega$ and the corresponding periodic cell, where the extracellular matrix and myocyte are denoted by $\Omega _{e}$ and $\Omega _{i}$, respectively. We identify the interface between the domains as $\Gamma :=\partial \Omega _{e}\cap \partial \Omega _{i}$ with corresponding unit normal ${\textbf{n}}$. This cell is shown in the asymptotic homogenization box of Fig. 1. We have that $|\Omega |=|\Omega _{i}|+|\Omega _{e}|$ is the domain volume and is equal to 1 since we assume we have a unit cube. Our periodic cell (cube) has periodic boundary conditions applied on all the faces, where we have assumed that the myocytes extend only in the z-axis direction.

### A.2 Porescale simulations

Here we present the results of the simulations that we carry out on the porescale microstructure shown in Fig. 5. Each of the myocytes and the extracellular matrix has an underlying porous structure. This means that we can calculate the poroelastic coefficients.

We assume that the porescale porous matrix of the myocyte has Young’s modulus $E_i=35$ and Poisson ratio $\nu _i=0.4$ and the porescale extracellular matrix has Young’s modulus $E_e=80$ and Poisson ratio $\nu _e=0.35$ where both phases are porous with porosities ranging from $\approx 2\% \, \text{to} \, \approx 50\%$.

To obtain the poroelastic parameters of the myocyte and the extracellular matrix, we must solve the typical cell problems of poroelasticity presented in Penta et al. (2020); Miller and Penta (2023); Dehghani et al. (2018).

The components of the elasticity tensors for the matrix and the myocyte are plotted versus increasing porosity.

Figure 11a and b shows that the 6 independent elastic parameters for both the myocyte and the extracellular matrix decrease with increasing porosity.

The Biot’s moduli for the individual phases are plotted versus the increasing porosity,

We note that these coefficients shown in Fig. 12a and b are indeed tending to $+\infty$ with increasing porosity; however, due to the constraints of the microstructure we have chosen for the underlying porous materials (see Fig. 5) we cannot exploit porosity beyond $50\%$.

The expressions for $\varvec{\alpha }_i$ and $\varvec{\alpha }_e$ are

96 $$\begin{aligned} \varvec{\alpha }_i=\phi {\textsf{I}}-\text{ Tr }({\mathbb {M}}_i) \quad \text{ and } \quad \varvec{\alpha }_e=\phi {\textsf{I}}-\text{ Tr }({\mathbb {M}}_e) \end{aligned}$$

where $\phi$ is the porosity of the phases and the fourth rank tensors ${\mathbb {M}}_i$ and ${\mathbb {M}}_e$ are so be found by solving the standard poroelastic cell problems found in Penta et al. (2020); Miller and Penta (2023); Dehghani et al. (2018). Due to the symmetry of the microstructure, $(\varvec{\alpha }_i)_{11}=(\varvec{\alpha }_i)_{22}$ and $(\varvec{\alpha }_e)_{11}=(\varvec{\alpha }_e)_{22}$. The Biot’s coefficients for the individual phases are plotted versus the increasing porosity.

The Biot’s coefficients shown in Fig. 13a and b for the matrix and the myocytes illustrate the standard behaviour of poroelastic media by tending to 1 with increasing porosity.

### A.3 Numerical simulations and meshing

In this appendix, we give an overview of the steps carried out in COMSOL Multiphysics to compute the results that we present throughout this work. We are using 2D simulations. The geometry that we are investigating is a long poroelastic myocyte embedded in a poroelastic (cube) matrix. The symmetries mean that the myocyte extends in the *z*-direction the length of the cell, and therefore, we are able to cut the plane, justifying the 2D simulations carried out.

For the electrical simulations, we have the cell problems (28a)–(28d) and (29a)–(29d) and our interface conditions (28d) and (29d) and are applied on the 2D line representation of the interface. For each boundary load given in (28d) and (29d), we compute the corresponding numerical solution of the diffusion-type problems (28a)–(28d) and (29a)–(29d). This is done using the finite element software COMSOL Multiphysics via the Transport of Diluted Species Module.

We use this software to compute the second rank tensors ${\textsf{R}}_i$, ${\textsf{R}}_e$, ${\textsf{Q}}_i$ and ${\textsf{Q}}_e$. Then once we have these results, they can be used in (81) to obtain the entries of the effective conductivity tensor ${\textsf{D}}$. These entries are used for the transversal and longitudinal conductances we plot in Fig. 4.

For the poroelastic simulations, we have the cell problems (47a)–(47d) and (48a)–(48d) and our interface conditions (47c) and (48c) are applied on the 2D line representation of the interface. For each boundary load given in (47c) and (48c) we compute a corresponding numerical solution of the elastic-type problems (28a)–(28d) and (29a)–(29d). This can be done using the finite element software COMSOL Multiphysics by employing the Structural Mechanics Module.

We use this software to compute the fourth rank tensors ${\mathbb {L}}_i$ and ${\mathbb {L}}_e$, as well as the second rank tensors $\varvec{\tau }_i$ and $\varvec{\tau }_e$. Then once we have these results, they can be used in (86) and (89) to obtain the entries of the effective elasticity tensor $\widetilde{{\mathbb {C}}}$ and the Biot’s modulus $\bar{{\mathcal {M}}}$. Once we have the complete tensor $\widetilde{{\mathbb {C}}},$ then we can use the components in the formulas for our elastic moduli $E_1$ and $E_3$ and take the shears directly from the tensor and plot in Figs. 6, 7, 8 and 9 and plot $\bar{{\mathcal {M}}}$ in Fig. 10.

We now describe how this process is carried out in COMSOL. This finite element software creates a mesh for our periodic cell $\Omega$. To do this it creates a surface mesh for the interfaces between the phases. Since in our case we are carrying out 2D simulations, we have just a line mesh. It then extends the line mesh into a two-dimensional one for the entire square periodic cell $\Omega$. This method is utilized as it allows for the interface conditions to be described by boundary pairs. This approach is very beneficial as it allows for a highly refined mesh on the interfaces (i.e. where the important interactions/physics occurs), and then, the bulk gets gradually coarser the further you move from the interface.

The cell problem (47a)–(47d) is driven by the stress jump condition on the matrix–myocyte interface; similarly, in (48a)–(48d) the driving force is the difference in the compressibility of the underlying phases. Due to these important conditions taking place on the interface, we require a sufficiently fine mesh here to ensure we obtain an accurate numerical solution. Therefore, to capture these areas where the important physics is taking place, our mesh in $\Omega$ is given additional refinement around the boundary pairs that representing the interfaces than in the rest of the domain. Due to the predefined features of COMSOL, we can use a sequence of increasingly refined meshes of $\Omega$ ranging from extremely coarse to extremely fine.

In cell problem (47a)–(47d), the stress balance Eqs. (47a) and (47b) are simple due to having zero volume forces; however, since the both the matrix $\Omega _{e}$ and the myocyte $\Omega _{i}$ are poroelastic, each has a constant, anisotropic elasticity tensor as an input value. We have that the stress jump and the continuity of the auxiliary tensors ${\mathcal {B}}_i$ and ${\mathcal {B}}_e$ given by (47c) and (47d) across the interface between the myocyte and the matrix are encoded by the conditions on each boundary pair. On the external boundary (edges of the square) $\partial \Omega ,$ we impose periodicity. This procedure means that the solution of the elastic-type problems will be unique up to a constant. We are not concerned by specifying the constant as it will disappear when the microscale derivatives of the solution are used to obtain ${\mathbb {L}}_{i}$ and ${\mathbb {L}}_{e}$. Even though we do not necessarily fixate on this constant, computationally we must have a unique solution in the periodic cell. We achieve this uniqueness via an additional constraint in COMSOL that sets the auxiliary displacement to zero at a single point in $\Omega$. This process fixes the constant that arises. The software COMSOL Multiphysics employs the principle of virtual work to implement these elastic-type problems in weak form. For cell problem (48a)–(48d), we carry out the same process with the difference in compressibility of the phases and the continuity of auxiliary vectors (48c) and (48d) encoded on the boundary pairs.

We remark that since we have the jump in stresses, the problem for the auxiliary variables ${\mathcal {B}}_i$ and ${\mathcal {B}}_e$ must be solved using the geometrical COMSOL feature assembly. The use of the union setting would mean that subdomains $\Omega _{i}$ and $\Omega _{e}$ would merge to form a simple union with continuity. This is not the case when modelling the myocardium. The assembly feature avoids this merging as you are able to retain the boundaries for each phase of the domain and allows for the correct application of the interface conditions. The same holds for the problem (48a)–(48d) and auxiliary tensors $\textbf{b}_i$ and $\textbf{b}_e$.

The fourth rank tensors ${\mathbb {L}}_i$ and ${\mathbb {L}}_e$ and the second rank tensors $\varvec{\tau }_i$ and $\varvec{\tau }_e$ are calculated by taking the microscale derivatives of the auxiliary variables ${\mathcal {B}}_i$ and ${\mathcal {B}}_e$, and $\textbf{b}_i$ and $\textbf{b}_e$, respectively. We can take simple linear derivatives of the entries of ${\mathcal {B}}_i$ and ${\mathcal {B}}_{e},$ and therefore, we can calculate all entries of the auxiliary fourth rank tensors ${\mathbb {L}}_{i}$ and ${\mathbb {L}}_{e}$ without error, similarly with second rank tensors $\varvec{\tau }_i$ and $\varvec{\tau }_e$. The entries of the effective elasticity tensor $\widetilde{{\mathbb {C}}}$ and the Biot’s modulus $\bar{{\mathcal {M}}}$ are then computed using (86) and (89) where we are able to take the averages without any additional errors occurring. All the steps carried out, including the finite element approximations for ${\mathcal {B}}_i$, ${\mathcal {B}}_e$, $\textbf{b}_i$ and $\textbf{b}_e$ to the computation of the effective elasticity tensor $\widetilde{{\mathbb {C}}}$ and Biot’s modulus $\bar{{\mathcal {M}}}$, are obtained from COMSOL Multiphysics via the integral post-processing tools. We note that the computational times for all these simulations are from 2 s to 90 s.
